# Alternative Pathway for Methyl Supply through the Coupling of SHMT1 and PEMT to Maintain Astrocytic Homeostasis in Parkinson's Disease

**DOI:** 10.1002/advs.202516794

**Published:** 2025-11-20

**Authors:** Yue‐Han Chen, Rong‐Xin Zhu, Nuo‐Xi Zhang, Ting‐Ting Sun, Xi‐Wei Zhang, Yu‐Jie Zhao, Ben‐Yu He, Hang Yao, Ren‐Hong Du, Lei Cao, Wen‐Bin Zhang, Wei‐Guo Liu, Yun Cai, Cong Wang, Gang Hu, Yao Wei, Yang Liu, Ming Lu

**Affiliations:** ^1^ Jiangsu Key Laboratory of Neurodegeneration, Department of Pharmacology Nanjing Medical University Nanjing 211116 China; ^2^ State Key Laboratory of Pharmaceutical Biotechnology Nanjing University Nanjing Jiangsu 210023 China; ^3^ Department of Pharmacology, School of Medicine Nanjing University of Chinese Medicine Nanjing Jiangsu 210023 China; ^4^ Changzhou Second People's Hospital, Changzhou Medical Center Nanjing Medical University Changzhou 213000 China; ^5^ Department of Neurology, Affiliated Nanjing Brain Hospital Nanjing Medical University No. 264 Guangzhou Road Nanjing Jiangsu 210029 China; ^6^ Basic Research Program of Jiangsu Jiangsu Province Innovation Center for Brain‐Inspired Intelligence Technology Nanjing Jiangsu 210023 China

**Keywords:** neuroexcitotoxicity, one‐carbon metabolism, parkinson's disease, SAM, SHMT1/PEMT

## Abstract

Serine Hydroxymethyltransferase 1 (SHMT1) plays a pivotal role in one‐carbon metabolism, facilitating the production of SAM. In this study, dysregulation of one‐carbon metabolism is reported in both Parkinson's disease (PD) patients and animal models, characterized by significantly downregulated expression of SHMT1. Astrocyte‐specific conditional knockout of Shmt1 decreased SAM level, exacerbated motor dysfunction, and dopaminergic neuronal loss in a PD mouse model. While SAM is conventionally generated through the one‐carbon cycle, the data indicate that, despite significant alterations in SHMT1, SAM remains unaffected while labeled ^13^C‐Serine. Intriguingly, isotopic labeling experiments revealed a significant association between SHMT1 and the production of PDME, an intermediate metabolite of the phosphatidylethanolamine methylation pathway. Consequently, PEMT is discovered as interacting with SHMT1. It is demonstrated that disruption of the interaction between SHMT1 and PEMT leads to SAM depletion, causing H3K4me1 hypomethylation, which in turn reduces the expression of Slc1a2 and Glul. As a result, decoupling of SHMT1 and PEMT in astrocytes ultimately exacerbates neuroexcitotoxicity and dopaminergic neuron loss in PD. Thus, the study elucidates the novel metabolic connection between SHMT1 and PEMT that links the astrocytic one‐carbon cycle and membrane phospholipid metabolism in PD.

## Graphical abstract

In Parkinson's disease, SHMT1 downregulation disrupts its interaction with PEMT in astrocytes, reducing SAM levels. This leads to H3K4me1 hypomethylation and decreased Slc1a2/Glul expression, ultimately exacerbating neuroexcitotoxicity and dopaminergic neuron loss. This study reveals a novel SHMT1‐PEMT link connecting one‐carbon and membrane phospholipid metabolism in PD pathogenesis.

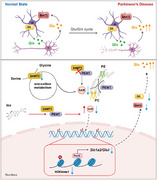

## 1 Introduction

Parkinson's disease (PD) is a progressive neurodegenerative disorder characterized by the selective loss of dopaminergic neurons of the Substantia nigra pars compacta (SNpc).^[^ ^1^ ^]^ Current evidence strongly suggests that PD results from complex interactions among genetic predisposition, environmental exposures, the misfolding and aggregation of α‐synuclein, mitochondrial impairment, oxidative stress, neuroinflammatory processes, and aberrant immune responses.^[^ ^2^ ^]^ However, the precise mechanisms driving neurodegeneration remain to be fully elucidated.

 One‐carbon metabolism comprises metabolic networks transferring one‐carbon units to biosynthetic pathways.^[^ ^3^ ^]^ Its core folate and methionine cycles sustain critical processes including nucleotide synthesis, amino acid homeostasis (glycine/serine/methionine), epigenetic regulation, and redox defense. Dysregulated one‐carbon metabolism is implicated in cardiovascular, oncological, immune, and neuropsychiatric pathologies.^[^ ^4^ ^]^ Aberrant flux through this pathway reduces methylation potential, exemplified by methylenetetrahydrofolate dehydrogenase (MTHFD2)‐dependent control of DNA/histone methylation in Th17 cells.^[^ ^5^ ^]^ Genetic ablation of methionine adenosyltransferase (MAT2a) in intestinal epithelia induces apoptosis—reversed by S‐adenosylmethionine (SAM) supplementation.^[^ ^6^ ^]^ Given its dual role in epigenetic‐metabolic crosstalk, one‐carbon metabolism is mechanistically linked to neurodegenerative disease pathogenesis.^[^ ^7^, ^8^ ^]^

Under physiological conditions, SHMT1 catalyzes serine and tetrahydrofolate conversion to 5,10‐methylenetetrahydrofolate and glycine in the folate cycle.^[^ ^9^ ^]^ In the CNS, SHMT1 depletion impairs one‐carbon metabolism, suppresses hippocampal neurogenesis, and induces cognitive deficits.^[^ ^10^ ^]^ Enhanced SHMT1 activity elevates one‐carbon flux, increases S‐adenosylmethionine (SAM) levels, and promotes histone methylation‐mediated chromatin remodeling, as demonstrated in pneumonia models.^[^ ^11^ ^]^ Paradoxically, methyltransferases like phosphatidylethanolamine N‐methyltransferase (PEMT) consume SAM to modify substrates, thereby regulating methylation dynamics—a process modulated by the SAM: S‐adenosylhomocysteine (SAH) ratio.^[^ ^12^ ^]^ Although SAM deficiency is known to drive histone hypomethylation and gene dysregulation in neurodegeneration, the role of SHMT in modulating SAM bioavailability and its implications for Parkinson's disease (PD) pathogenesis remain unexplored.

Our study identified dysregulated one‐carbon metabolism in the SNpc of both PD mouse models and patients. Decreased SHMT1 expression correlated with PD motor symptom severity. Critically, disrupted SHMT1‐PEMT interaction reduced SAM levels, causing histone hypomethylation at the EAAT2/GS promoter. This epigenetic dysregulation impaired glutamate‐glutamine cycle homeostasis. The natural compound Isoorientin enhanced SHMT1‐PEMT binding, exerting neuroprotection. Collectively, the SHMT1/PEMT axis represents a novel regulatory pathway for SAM biosynthesis, modulating neuronal excitability and offering a therapeutic target for PD.

## 2 Results

### 2.1 One‐Carbon Metabolism Key Enzyme SHMT1 is Associated with PD Progression

**Figure 1:**
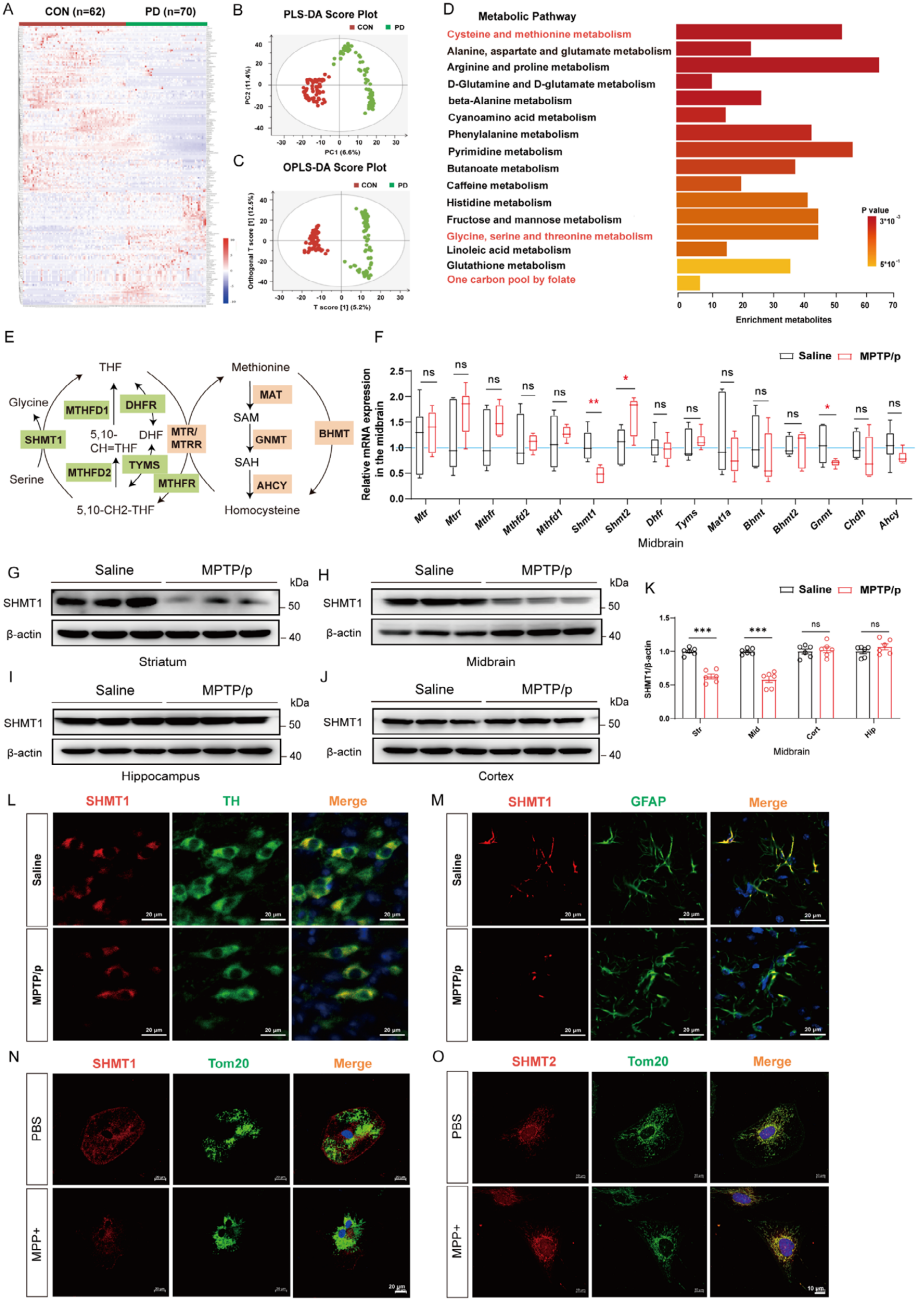
The one‐carbon metabolism is reprogrammed in both PD mice and patients. A) Metabolite heatmap of peripheral serum samples from 70 PD patients and 62 healthy controls. B,C) Data quality control used by Partial Least Squares‐Discriminant Analysis (PLS‐DA) and orthogonal partial least squares discriminant analysis (OPLS‐DA); Green: PD patient group. Red: healthy control group. D) Enrichment of metabolic pathway analysis. The color scheme represents the P value against the control group. Red letters labeled the corresponding pathway included one‐carbon metabolism. E) The schematic diagram illustrates the key metabolite enzyme in one‐carbon metabolism. It was independently reproduced and reanalyzed using publicly accessible data (doi:10.1146/annurev‐animal‐020518‐115206). F) The mRNA levels of the one‐carbon metabolism pathway were validated in the midbrain of MPTP/p mice (n = 6). G–K) The protein levels and statistical analysis of SHMT1 across different brain regions (midbrain, striatum, cortex, and hippocampus) in MPTP/p mice, n = 6. L) Representative co‐localization images of SHMT1 (red) and Tom 20 (green) in the MPP^+^ model. Scale bar, 20 µm. M) Representative co‐localization images of SHMT2 (red) and Tom 20 (green) in the MPP^+^ model. Scale bar, 10 µm. N) Representative co‐localization images of SHMT1 (red) and dopaminergic neuron marker TH (green) in the midbrain of the MPTP model. Scale bar, 20 µm. O) Representative co‐localization images of SHMT1 (red) and astrocyte marker GFAP (green) in the midbrain of the MPTP model. Scale bar, 20 µm. Data were analyzed using one‐way ANOVA, followed by Tukey post‐hoc tests, and presented as mean ± SEM. ^*^ *p* < 0.05, ^**^ *p* < 0.01, ^***^ *p* < 0.001, ns: no significance.

To characterize metabolic diversity in the brain, the genes, and pathways underlying PD progression, we performed untargeted metabolomics analysis on peripheral serum samples from 70 PD patients and 62 healthy controls (**Figure** **1**A). Partial Least Squares‐Discriminant Analysis (PLS‐DA) and orthogonal partial least squares discriminant analysis (OPLS‐DA) analysis illustrated a significant separation between these two groups (Figure 1B,C). To gain a comprehensive view of the untargeted metabolomics data, KEGG enrichment analysis was performed on the differential metabolites. As shown in Figure 1D, we identified involvement of the cystine and methionine metabolism pathway, the glycine, serine, and threonine metabolism pathway, and the one‐carbon pool by folate in the pathophysiological state of PD patients. Notably, amino acids enriched in one‐carbon metabolism were significantly altered, while alterations in taurine and uric acid were consistent with a previous report (Figure S1, Supporting Information). Therefore, we concluded that one‐carbon metabolism is reprogrammed in PD patients (Figure 1E). Subsequently, our focus shifted to 15 genes within the one‐carbon metabolism pathway. Validation through RT‐qPCR highlighted *Shmt1* as the most significantly differentially expressed gene among the one‐carbon metabolism pathway (Figure 1F). Notably, the protein levels of SHMT1 in the midbrain and striatum exhibited a consistent decline in the MPTP/p mouse model (Figure 1G,H). Importantly, this decline did not influence the protein levels of SHMT1 in the cortex and hippocampus, indicating a brain region‐specificity of SHMT1 in the pathology of PD (Figure 1I–K). Furthermore, correlation analysis revealed a positive relationship between SHMT1 expression and PD motor symptoms in the MPTP mouse model. (Figure S2, Supporting Information). To identify the cell type expressing SHMT1, we examined its expression in brain slices using multi‐immunohistochemical fluorescent staining. In the midbrain, SHMT1 was expressed in both neurons and astrocytes in the saline group, whereas it was notably decreased in astrocytes rather than neurons in the MPTP/p‐treated group, indicating that astrocytic SHMT1 may play an important role in PD (Figure 1L,M). Moreover, SHMT1 was mainly localized in the cytoplasm but not in the mitochondria (Figure 1N,O; Figure S3, Supporting Information). Collectively, our findings suggest the impaired one‐carbon metabolism and decreased SHMT1 expression in both PD mice and patients, thereby highlighting the important role of SHMT1 in PD pathology.

### 2.2 Astrocytic Knockout of *Shmt1* Aggravates Motor Symptom Dysfunction and DA Neuronal Loss in the MPTP/p Mouse Model

As indicated by the results, there is a close correlation between SHMT1 expression and PD motor symptoms. Next, we investigated the role of SHMT1 in regulating motor symptoms by performing behavioral tests using the MPTP/p mouse model. First, we constructed a lentivirus (LV) for the overexpression of *Shmt1* and microinjected it into the substantia nigra (Figure S4A,B, Supporting Information). The area of viral infection was validated through immunofluorescence staining of zsGreen, confirming successful infection (Figure S4C, Supporting Information). Subsequently, behavioral tests were conducted. In the open field test, overexpression of *Shmt1* resulted in increased distance traveled by PD mice (Figure S4D,E, Supporting Information). Furthermore, in the rotarod test, overexpression of *Shmt1* prolonged the latency to fall following MPTP/p treatment (Figure S4F, Supporting Information). Notably, in the gait trace test, the stride length was decreased, and the movement speed change rate was prolonged when *Shmt1* was overexpressed prior to MPTP/p treatment (Figure S4G–I, Supporting Information). Meanwhile, overexpression of *Shmt1* rescued TH‐positive neurons (Figure S4J,K, Supporting Information) in the SNpc. Consistently, the protein level of TH was downregulated in the MPTP/p groups, whereas overexpression of *Shmt1* rescued the decline (Figure S4L,M, Supporting Information). These results suggest that overexpression of *Shmt1* ameliorates PD motor symptoms and DA neuronal loss.

**Figure 2:**
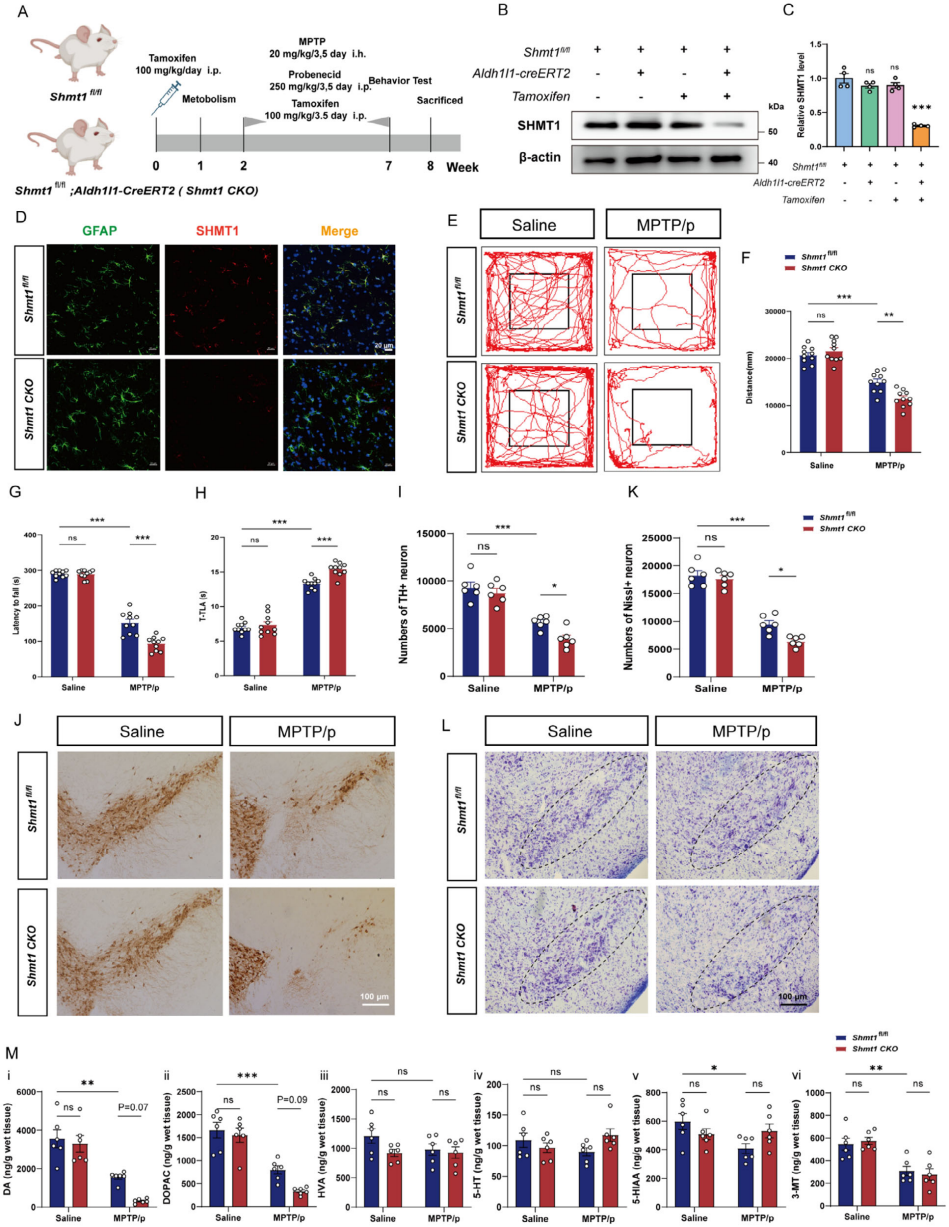
Knockout of astrocytic *Shmt1* aggravates the dyskinesia and loss of dopaminergic neurons in the MPTP/p mouse model. A) Schematic model of the experimental procedure. B,C) Representative immunoblot images and quantification of SHMT1 while conditionally *Shmt1* is knocked out in astrocytes. n = 4. Data were analyzed by an unpaired t‐test. ^***^ *p* < 0.001, ns: no significance. D) Representative images showing the expression of SHMT1 in the SNpc. Enlarge vision: Scale bars, 20 µm. Motor function performance of mice detected in *Shmt1* ^fl/fl^ (Saline, n = 10 mice; MPTP/p, n = 10 mice) or *Shmt1* ^fl/fl^ *; Aldh1l1‐CreERT2* (Saline, n = 10 mice; MPTP/p, n = 10 mice) in (E,F) the open field test, G) the rotarod test, and H) the pole test. I,J) Representative immunohistochemical images and quantification of TH‐positive neurons in the SNpc region, n = 6. Scale bar, 100 µm. K,L) Representative immunohistochemical images and quantification of Nissl‐positive neurons in the SN region, n = 6. Scale bar, 100 µm. M) The levels of DA, DOPAC, NE, HVA, 5‐HT, 5‐HIAA, and 3‐MT in the striatum were detected by HPLC, n = 6. Data were analyzed using two‐way ANOVA, followed by Tukey post‐hoc tests, and presented as mean ± SEM. ^*^ *p* < 0.05, ^**^ *p* < 0.01, ^***^ *p* < 0.001, ns: no significance.

Given the predicted significant role of astrocytic SHMT1, we constructed gene‐knockout mice with astrocyte‐specific deletion of *Shmt1* utilizing Cre‐loxP systems and implemented the MPTP/p model to elucidate the specific role of astrocytic SHMT1 in PD progression (**Figure** **2**A; Figure S5, Supporting Information). The efficiency of conditional knockout was validated (Figure 2B–D). Immunofluorescence staining of SHMT1 and GFAP confirmed the successful deletion of astrocytic *Shmt1* in brain slices (Figure 2D). As shown in Figure 2E–H, *Shmt1* ^fl/fl^ *; Aldh1l1‐CreERT2* mice performed an aggravated phenotype in the MPTP/p mouse model, characterized by decreased movement distance, shortened latency to fall, and prolonged time. Notably, astrocytic knockout of *Shmt1* resulted in increased DA (Figure 2I,J) and Nissl neuronal loss in the MPTP/p mouse model (Figure 2K,L). Analysis of neurotransmitter levels in the striatum by HPLC revealed that astrocytic knockout of *Shmt1* significantly decreased the levels of dopamine and its metabolites (DOPAC and HVA) in MPTP/p‐treated WT mice (Figure 2M). In conclusion, these findings highlight that overexpression of astrocytic *Shmt1* represents a promising neuroprotective effect to decelerate the progression of PD.

### 2.3 SHMT1 Facilitates the Production of SAM through the Alternative Way of the Demethylation of PC to PE

**Figure 3:**
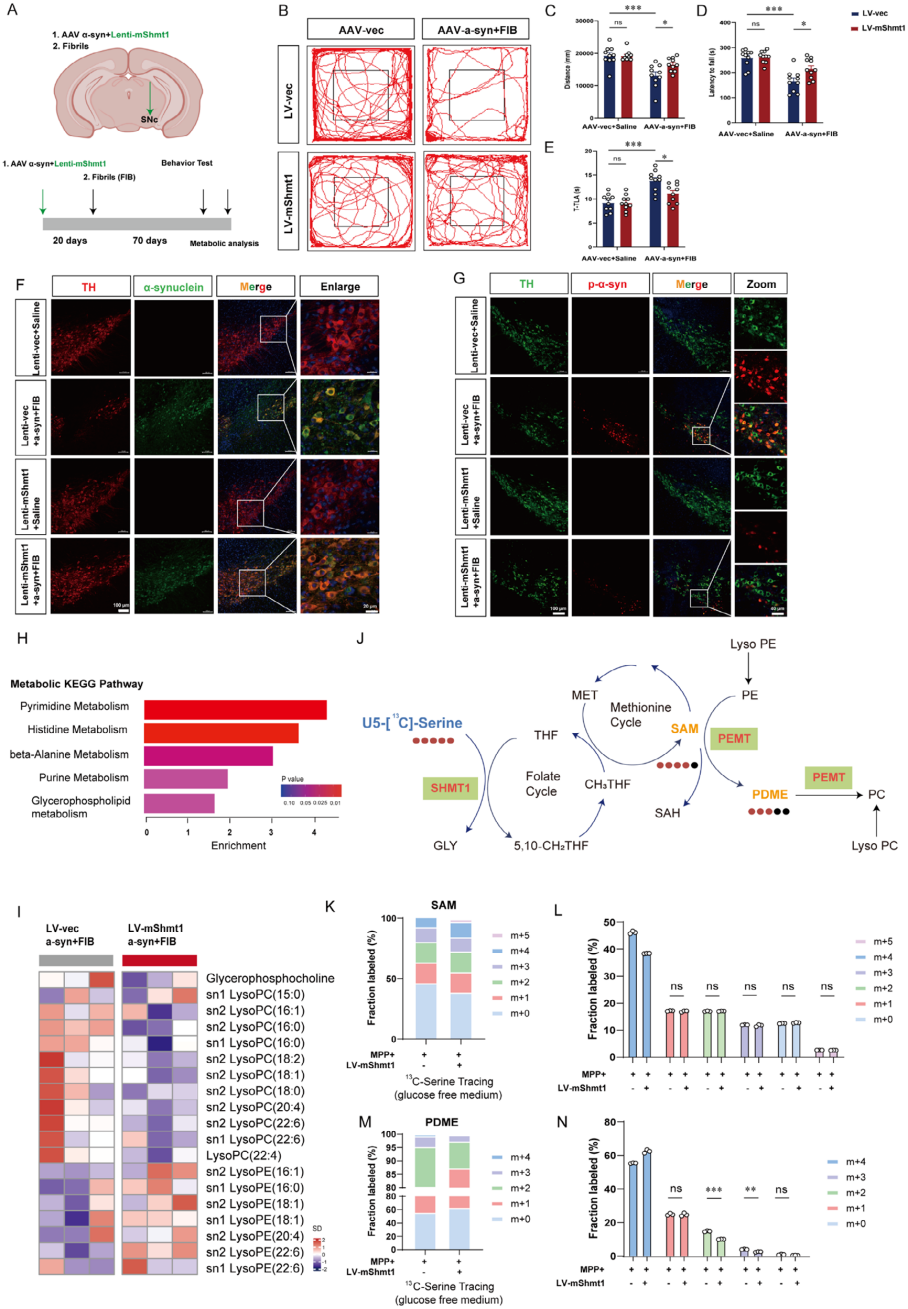
*Shmt1* promotes the transition of phospholipid peroxidation to a neuroprotective phenotype in the PFF mouse model. A) Schematic diagram of the experimental procedure and Lenti‐m*Shmt1*/AAV‐α‐synuclein plus fibrils injection site. Panel A schematic was generated in BioRender (license no. *TU28UVIL4Z*) and refined with Adobe Illustrator 2022 (v26.0.0). B) Representative images showing the LV injection site in the SNpc region. Motor function performance of mice injected with Lenti‐vec/AAV‐α‐synuclein plus fibrils (Lenti‐vec, n = 10 mice; Lenti‐vec+PFF, n = 10 mice) or Lenti‐m*Shmt1*/AAV‐α‐synuclein plus fibrils (Lenti‐m*Shmt1*, n = 10 mice; Lenti‐m*Shmt1*+PFF, n = 10 mice) in (C) the open field test, D) the rotarod test, and E) the pole test. F) Representative co‐localization images of α‐synuclein (green) and TH (red) in the PFF mouse model. Scale bar, 100 µm. Enlarge vision; Scale bar, 20 µm. G) Representative co‐localization images of p‐α‐synuclein (red) and TH (green) in the PFF mouse model. Scale bar, 100 µm. Enlarge vision; Scale bar, 20 µm. H) KEGG enrichment pathways in the striatum of the PFF mouse model. I) Heatmap showing the differentially expressed metabolites in the Glycerophospholipid metabolism pathway. J–L) Analysis of ^13^C‐labeled metabolites by liquid chromatography‒mass spectrometry (LC‒MS) after astrocytes overexpressing the LV‐vector or LV‐SHMT1 were incubated with ^13^C‐serine for 12 h. The schematic diagram illustrates the conversion of U‐^13^C‐serine into SAM (n = 3 biologically independent samples). The schematic diagram (J) was independently reproduced and reanalyzed using publicly accessible data. (doi:10.1146/annurev‐animal‐020518‐115206.). M,N) A similar analysis to metabolite is PDME. Data were analyzed using two‐way ANOVA, followed by Tukey post‐hoc tests, and presented as mean ± SEM. ^*^ *p* < 0.05, ^**^ *p* < 0.01, ^***^ *p* < 0.001, ns: no significance.

To evaluate the potential role of *Shmt1* on the pathology of PD, we adapted another accelerated model involving the co‐injection of target protein virus and α‐synuclein overexpressed AAV as previously reported^[^ ^13^ ^]^ (**Figure** **3**A). As indicated in Figure 3B,C, overexpression of *Shmt1* led to a significant increase in the distance traveled by PD mice during the open field test. Likewise, overexpression of *Shmt1* prolonged the time to drop off in the rotarod test and reduced the time taken in the pole test (Figure 3D,E). Meanwhile, we evaluated the impact of *Shmt1* on α‐synuclein aggregation within this mouse model. Notably, immunofluorescence staining revealed a correlation between the loss of DA neurons and the accumulation of p‐α‐synuclein in the substantia nigra, confirming the successful establishment of the PD mouse model (Figure 3F,G). To gain a comprehensive understanding of *Shmt1*’s role in the progression of PD, we collected the striatal tissues and conducted untargeted metabolomics. Metabolic KEGG pathway analysis suggested significant enrichment in the Glycerophospholipid pathway (Figure 3H). Then we analyzed the metabolites in the Glycerophospholipid pathway, and we found that LysoPC was downregulated and LysoPE was significantly decreased in the striatum of the PFF mouse model (Figure 3I). As illustrated in Figure 3J, SAM synthesis originates from the methionine cycle, which is derived from the folate cycle. Using LC‐MS‐based ^13^C‐serine stable isotope tracing, we found no increase in M+3 SAM levels, suggesting that SHMT1 does not directly contribute to SAM biosynthesis (Figure 3K,L). Moreover, stable isotope‐based metabolic flux data revealed an intermediate named Phosphatidyl‐N, N‐dimethylethanolamine (PDME), generated during the demethylation of phosphatidylcholine (PC) to regenerate phosphatidylethanolamine (PE). In this process, PEMT transfers a methyl group from the choline headgroup of PC to the cofactor SAH, forming SAM.^[^ ^14^ ^]^ As expected, we observed a significant downregulation of PDME when SHMT1 is overexpressed (Figure 3M,N). In conclusion, these results indicate that PEMT acts as a bridge in SHMT1 and SAM biosynthesis.

### 2.4 SHMT1 Overexpression Enhances the Synthesis of S‐Adenosyl Methionine (SAM), Leading to Increased Monomethylation of Histone H3 at Lysine 4

**Figure 4:**
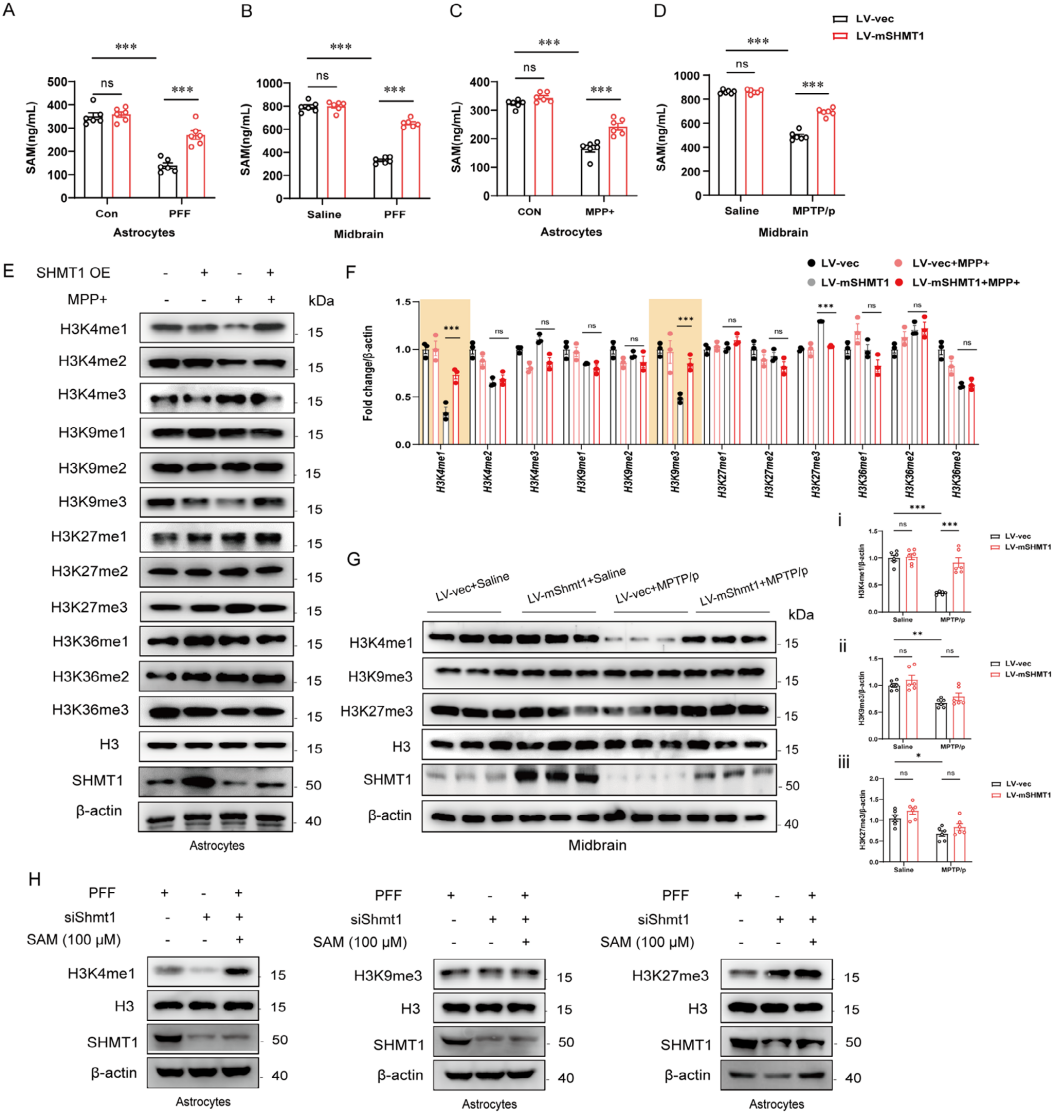
SHMT1‐mediated S‐adenosyl methionine (SAM) promotes the histone H3K4me1 methylation. A,B) The concentration of SAM was detected in the astrocytes and midbrain of the PFF mouse model. C,D) The concentration of SAM was detected in the astrocytes of the MPP^+^ model and the midbrain of MPTP/p mouse model, while SHMT1 was overexpressed. E,F) Representative immunoblot images and quantification of histone methylation H3K4me1/2/3, H3K9me1/2/3, H3K27me1/2/3, and H3K36me1/2/3 in astrocytes (n=3). G) Representative immunoblot images and quantification of histone methylation H3K4me1, H3K9me3, and H3K27me3 in the midbrain (n = 3). H) Representative immunoblot images of histone methylation H3K4me1, H3K9me3, and H3K27me3 while Shmt1 is knocked down and supplemented with SAM in astrocytes. Data were analyzed using two‐way ANOVA, followed by Tukey post‐hoc tests, and presented as mean ± SEM. ^*^ *p* < 0.05, ^**^ *p* < 0.01, ^***^ *p* < 0.001, ns: no significance.

SHMT1 serves as a pivotal enzyme in the one‐carbon metabolism pathway.^[^ ^11^ ^]^ The ^13^C‐serine stable isotope tracing showed that SHMT1 does not interfere with the biosynthesis of SAM. However, the relationship between SAM production and SHMT1 remains unknown. Therefore, we prepared the MPTP/p and PFF mouse models, and analysis of SAM levels revealed a notable reduction in SAM levels in the PD model compared to the mock counterparts, both in vitro and in vivo, consistent with the untargeted metabolomics data in PD patients (Figure S1A, Supporting Information). The ELISA kit demonstrated that overexpression of *Shmt1* rescued the decline in SAM levels (**Figure** **4**A–D). To further investigate the role of SAM, we conducted experiments using the PFF model by injecting fibrils into the striatum, a well‐established model reported previously.^[^ ^13^ ^]^ We successfully validated the model in our study (Figure S6, Supporting Information). In the open‐field test, SAM treatment increased movement distance in the PFF mouse model (Figure S7A–C, Supporting Information). Meanwhile, SAM treatment extended the latency to fall in the rotarod test and improved performance in the pole test (Figure S7D,E, Supporting Information). Likewise, in the gait trace test, SAM treatment boosted the front stance width and movement speed change rate in the PFF mouse model (Figure S7F–H, Supporting Information). Additionally, SAM treatment reduced the loss of DA neurons in the nigrostriatal pathway, as demonstrated by immunofluorescence staining for TH in the SNpc (Figure S7I, Supporting Information). These results indicate that SAM supplementation mitigates the loss of DA neurons and improves motor symptoms in the PFF mouse model.

To elucidate the specific downstream methylated proteins, we further investigated the histone methylation level while *Shmt1* was overexpressed. Immunoblotting and immunofluorescence showed that H3K4me1 and H3K9me3 were upregulated, and H3K27me3 was significantly downregulated in MPP^+^‐treated astrocytes following overexpression of *Shmt1* (Figure 4E,F; Figure S8, Supporting Information). Meanwhile, immunoblotting in the midbrain highlighted H3K4me1 as the most significantly differentially expressed methylated histone among these proteins (Figure 4G). Notably, H3K4me1 level diminished upon *Shmt1* knockdown but was enhanced with SAM supplementation in astrocytes (Figure 4H). Our findings demonstrate that SHMT1 facilitates SAM production, thereby intensifying histone methylation at H3K4me1 in PD.

### 2.5 SHMT1 Sustains H3K4me1 Histone Methylation to Suppress Neuroexcitotoxicity in Parkinson's Disease

**Figure 5:**
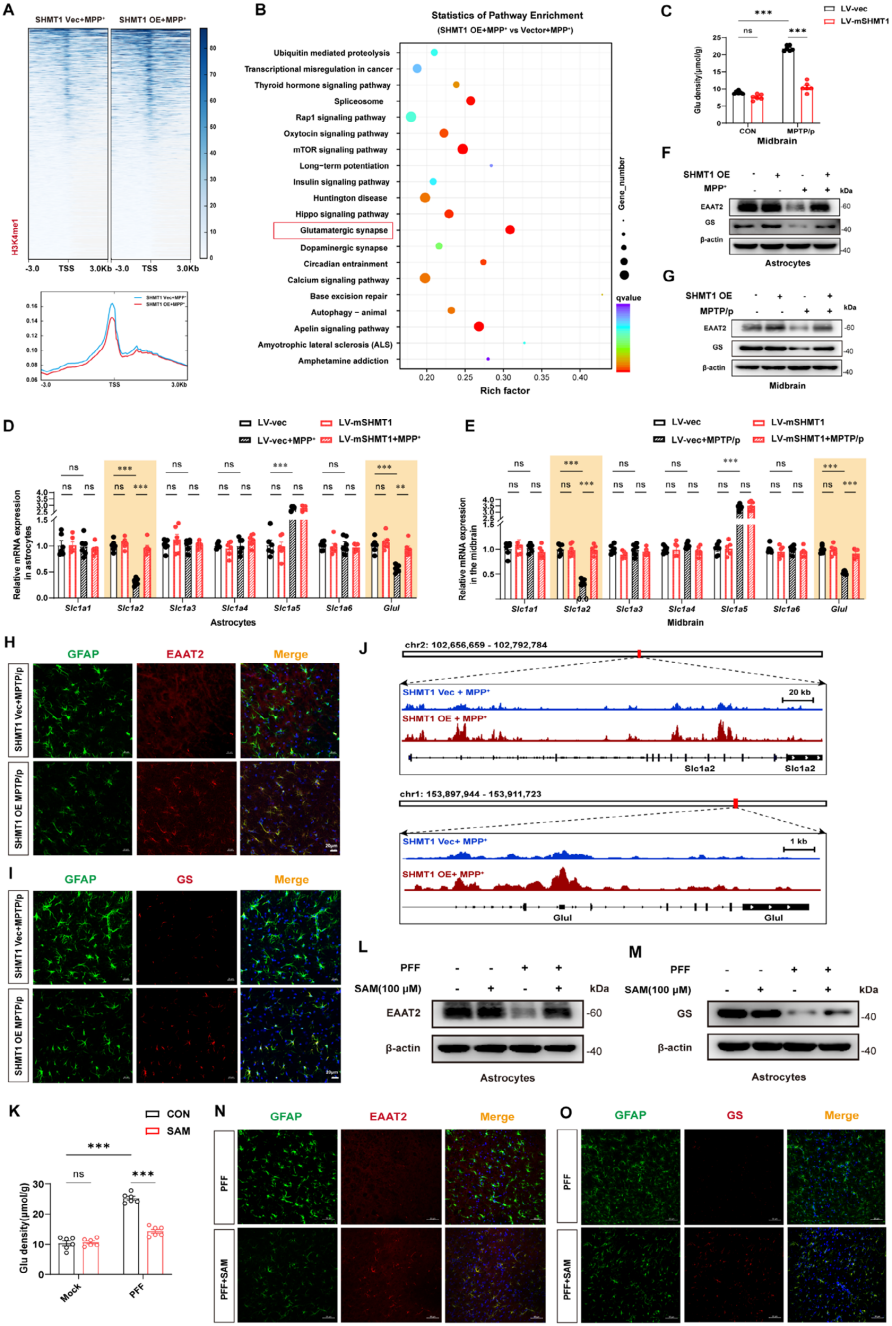
Genome‐wide analysis of the transcriptional consequences of H3K4me1 in SHMT1‐overexpressing astrocytes. A) Genome‐wide heatmaps of H3K4me1 ChIP‐seq TSS peak‐centered signal in vector and SHMT1‐overexpressing astrocytes, ordered by signal strength (upper panel). Read density metaplots showing average reads per kilobase per million (RPKM)‐normalized signals in vector and SHMT1‐overexpressing astrocytes (lower panel). B) KEGG enrichment pathway analysis using unfiltered gene expression data of the *Shmt1* overexpressed group and the controls. C) The glutamate (Glu) levels are detected in the striatum in the *Shmt1* overexpressed group and the controls. Differentially expressed genes in the Glutamatergic synapse pathways were validated by RT‐qPCR in (D) the astrocytes and E) the midbrain. The protein levels and analysis of F) EAAT2 and G) GS are examined by immunoblotting, while *Shmt1* is overexpressed in astrocytes. H) Representative co‐localization images of EAAT2 (red) and astrocyte marker GFAP (green) in the MPTP/p mouse model. Scale bar, 20 µm. I) Representative co‐localization images of GS (red) and astrocyte marker GFAP (green) in the MPTP/p mouse model. Scale bar, 20 µm. J) The chromatin track map of *Slc1a2* and *Glul* was performed in the IGV software. K) The glutamate (Glu) levels are detected in the striatum while SAM was supplemented in the PFF model (n=6). The protein levels of L) EAAT2 and M) GS are examined by immunoblotting while SAM was supplemented in the PFF model. N,O) Representative co‐localization images of EAAT2/GS and astrocyte marker GFAP while SAM was supplemented in the PFF model. Scale bar, 50 µm. Data were analyzed using two‐way ANOVA, followed by Tukey post‐hoc tests, and presented as mean ± SEM. ^*^ *p* < 0.05, ^**^ *p* < 0.01, ^***^ *p* < 0.001, ns: no significance.

Building on our previous findings that SHMT1 facilitates histone methylation of H3K4me1, we conducted the H3K4me1 CHIP‐seq to investigate its effects on the downstream pathway. The heatmap showed a panel of genes significantly upregulated when Shmt1 was overexpressed in astrocytes (**Figure** **5**A). KEGG enrichment analysis indicated a strong enrichment in the Glutamatergic synapse pathways (Figure 5B). As is known, Glutamate is the major excitatory neurotransmitter in the central nervous system (CNS). Once released into the synaptic cleft, glutamate acts on glutamate receptors to mediate fast excitatory synaptic transmission. In glia cells, glutamate is converted to glutamine, which is then transported back to the presynaptic terminal and converted back to glutamate.^[^ ^15^ ^]^ We first assessed the levels of glutamate in the striatum. Results showed that overexpression of *Shmt1* can greatly reduce the level of glutamate (Figure 5C). Consequently, we screened the genes involved in the glutamate‐glutamine cycle by QT‐PCR. Results showed that overexpression of *Shmt1* significantly promoted the expression of *Slc1a2* (EAAT2) and *Glul* (GS) in astrocytes (Figure 5D) and striatum (Figure 5E). Consistently, immunoblotting data suggested that both EAAT2 and GS were highly upregulated following overexpression of *Shmt1* (Figure 5F,G; Figure S9, Supporting Information). Immunofluorescence staining confirmed that both EAAT2 and GS were distributed in astrocytes and upregulated in the *Shmt1*‐overexpression group (Figure 5H,I). Moreover, track mapping indicated that the promoters of *Slc1a2* (EAAT2) and *Glul* (GS) *in* the glutamate‐glutamine cycle were highly enriched (Figure 5J). As it is speculated that the homeostasis of the glutamate‐glutamine cycle is regulated by methylation, after treatment with SAM, interestingly, the level of glutamate is decreased (Figure 5K), and the expression of *Slc1a2* (EAAT2) and *Glul* (GS) is increased in the striatum (Figure 5L,M). Consistently, immunofluorescence staining confirmed that SAM alleviated the activation of astrocytes and enhanced both EAAT2 and GS expression colocalized in astrocytes in the midbrain (Figure 5N,O).

Furthermore, to investigate the potential transcription factors involved in the motif reverse peaks of *Slc1a2* and *Glul*, *Foxl2* was selected by the Wenn diagram (Figure S10A, Supporting Information). ChIP assays were further performed to validate whether *Foxl2* directly regulates EAAT2 and GS transcription as a transcription factor. The results showed that *Foxl2* was recruited to the promoter region of EAAT2 and GS (Figure S10B–D, Supporting Information). *Foxl2* overexpression increased *Slc1a2* and *Glul* luciferase activity in HEK293T cells through transfection of the reporter system (Figure S10E–G, Supporting Information). Moreover, we conducted H3K4me1 ChIP‐qPCR on the EAAT2 and GS promoters under the conditions of SHMT1 overexpression and PEMT knockdown treatment in the present revision. The results confirmed that the enhancer activation is dependent on the SHMT1‐PEMT coupling (Figure S10H–K, Supporting Information). Collectively, these results demonstrate that SHMT1‐mediated SAM modulates histone monomethylation at H3 lysine 4 residue, thereby regulating the *Foxl2‐* EAAT2‐GS signaling pathway.

### 2.6 Isoorientin Enhanced the Interaction between SHMT1 and PEMT to Exert Neuroprotection in PD

**Figure 6:**
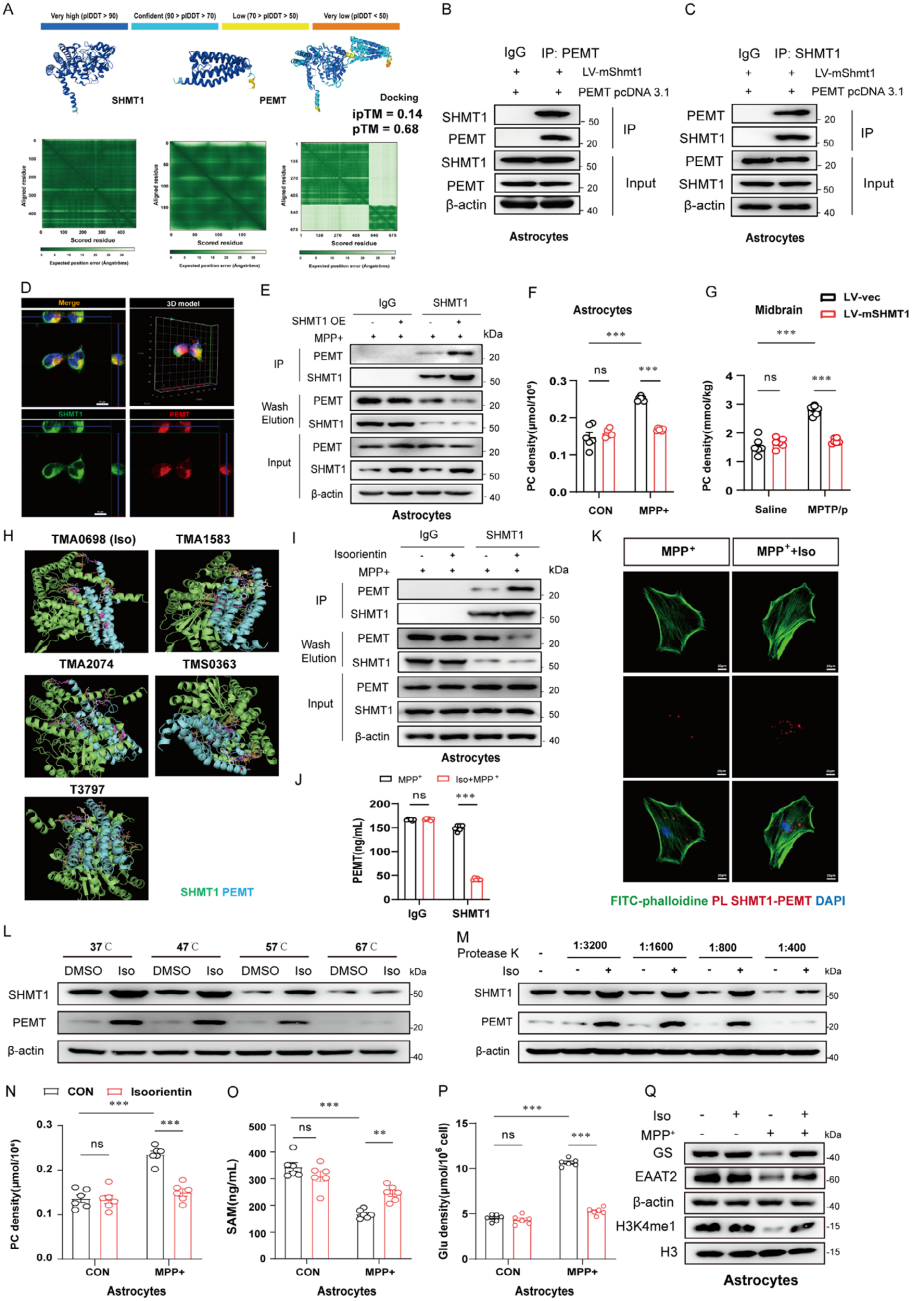
Formation of the SHMT1‐PEMT complex promotes SAM biosynthesis via upregulation of histone methylation H3K4me1. A) Interaction prediction between the SHMT1 and PEMT via the AlphaFold protein structure database. B,C) Cell lysates were immunoprecipitated with anti‐PEMT antibody or anti‐SHMT1 antibody, and then the samples were analyzed by immunoblotting. D) Immunofluorescent staining for SHMT1 and PEMT in HEK‐293T cells. Green: β‐SHMT1; Red: PEMT; Blue: DAPI. Scale bar: 10 µm. E) Cells were overexpressed with SHMT1, followed by cell lysates that were immunoprecipitated with anti‐SHMT1 antibody, and then the samples were analyzed by immunoblotting. F,G) The levels of Phosphatidylcholine (PC) are detected while SHMT1 is overexpressed in the astrocytes and the midbrain. n = 6. H) Active pockets in the combination of SHMT1 and PEMT were docked with 5088 drugs in the natural compound library. Five drugs (TMA0698‐Iso, TMA1583, T3797, TMA2074, and TMS0363) were selected for further study. I) Cells were pretreated with Isoorientin (10 µm) and MPP^+^ (500 µm), followed by immunoprecipitation of cell lysates with an anti‐SHMT1 antibody. The samples were then analyzed by immunoblotting. J) ELISA detected the relative PEMT levels in the washing solution of IP samples. K) Primary astrocytes were pretreated with MPP^+^ (500 µm) or Isoorientin (10 µm). Red spots indicate the SHMT1 and PEMT proximity ligation signals. Scale bar, 20 µm. L) CETSA was performed as described in the Experimental Section. The stabilizing effects of Isoorientin on SHMT1/PEMT at different temperatures were evaluated by Western blot analysis. M) DARTS was performed as described in the Experimental section. The protease stability of SHMT1/PEMT by Isoorientin was evaluated by Western blot analysis. N) The levels of Phosphatidylcholine (PC) were detected in the astrocytes pretreated with Isoorientin (10 µm). O,P) The levels of SAM and Glu were detected in the astrocytes pretreated with Isoorientin (10 µm). Q) Immunoblot analysis of GLUL, EAAT2, and H3K4me1 in the astrocytes. n=3. Data were analyzed using two‐way ANOVA, followed by Tukey post‐hoc tests, and presented as mean ± SEM. ^*^ *p* < 0.05, ^**^ *p* < 0.01, ^***^ *p* < 0.001, ns: no significance.

Our previous data suggested that SHMT1 does not directly promote the SAM biosynthesis; significant downregulation of PDME upon SHMT1 overexpression demonstrates that PEMT bridges SHMT1 to SAM biosynthesis. Therefore, we speculated that the elevated accumulation of SAM results from the binding of SHMT to PEMT. As shown in **Figure** **6**A, visual docking predicts the interaction between SHMT1 and PEMT. Co‐immunoprecipitation (Co‐IP) experiments were conducted to validate the SHMT1‐PEMT complex (Figure 6B,C). Following the co‐transfection, PEMT exhibited colocalization with SHMT1 within HEK293T cells (Figure 6D). Moreover, in the MPP^+^ stimulated model, overexpression of SHMT1 significantly decreased PEMT levels in the washing solution samples due to the combination (Figure 6E). Since PEMT catalyzes the conversion of DMPE to PC, we observed that the level of PC was significantly decreased in both astrocytes and the midbrain (Figure 6F,G). Therefore, the SHMT1‐PEMT complex is involved in shifting PC toward PE, thereby leading to increased accumulation of SAM. To clarify the functional coupling between SHMT1 and PEMT, we knocked down PEMT in primary astrocytes and detected the level of SAM, Glutamate, H3K4me1, and the subsequent upregulation of EAAT2/GS expression, in the presence of SHMT1 overexpression. We found PEMT deficiency partially reversed the neuroprotective benefits conferred by SHMT1 upregulation against excitotoxicity in the MPP^+^‐induced PD model (Figure S11, Supporting Information). Given our findings regarding the interaction between SHMT1 and PEMT, we aim to find a natural product‐derived compound that targets the SHMT1‐PEMT complex. Therefore, we performed virtual screening using a library of 5088 natural compounds. Five drug candidates (TMA0698, TMA1583, T3797, TMA2074, and TMS0363) were identified that likely promote the interaction of SHMT1 and PEMT (Figure 6H). It is suggested that Iso binds to the loop region of PEMT, stabilizing the conformation of the loop region and forming hydrogen bonds with SHMT1. Our results demonstrated that the highest rated drug, TMA0698 (Isoorientin, Iso), remarkably enhanced the combination of SHMT1 and PEMT by Co‐IP (Figure 6I). As expected, Iso‐induced binding of SHMT1 to PEMT led to a great decrease in the free form of PEMT. Consistently, the ELISA assay validated that PEMT levels were significantly decreased in the washing solution samples from the Isoorientin‐treated group (Figure 6J). Moreover, the proximity ligation assay illustrated that Iso can strongly enhance the combination between SHMT1 and PEMT (Figure 6K; Figure S10L,M, Supporting Information). To confirm whether Isoorientin is directly bound to the SHMT1/PEMT complex, we conducted a cellular thermal shift assay (CETSA) and drug affinity responsive target stability (DATRS) assay. First, we collected the Co‐IP samples contain the SHMT1‐PEMT complex. Then we incubated the lysates with or without 10 µm Iso for 10 min. Interestingly, we found that Iso significantly promoted SHMT1 and PEMT protein stability (Figure 6L). Consistent with the results, we then incubated the lysate with various concentrations of protease K for 10 min at room temperature, and found that Iso could bind to and stabilize SHMT1‐ PEMT complex in vitro (Figure 6M). Overall, results showed that Isoorientin treatment strengthened the thermal and protease stability of SHMT1/PEMT. As shown in Figure 6N,O, the levels of PC were greatly decreased, while SAM production increased in the Iso‐treated group. Therefore, the H3K4me1 was subsequently upregulated, accompanied by decreased levels of Glutamate and increased EAAT2/GS (Figure 6P,Q). Overall, these results suggest that Isoorientin enhances the interaction between SHMT1 and PEMT in vitro.

**Figure 7:**
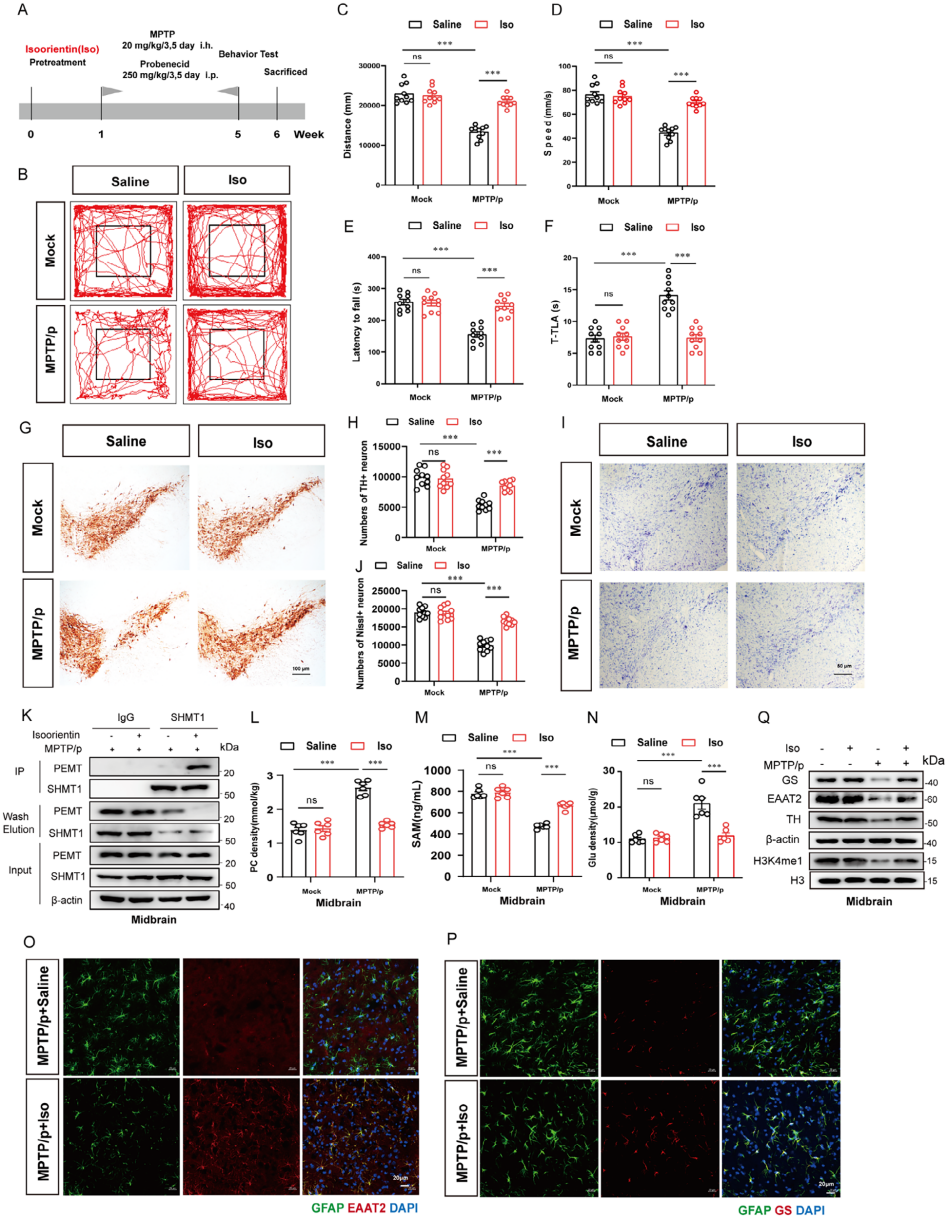
Isoorientin promotes the interaction of SHMT1 and PEMT to mediate the neuroprotective effect in the PD mouse model. A) Schematic diagram of the experimental procedure. Motor function performance of mice was detected (Saline, n = 10 mice; MPTP/p, n = 10 mice; Isoorientin, n = 10 mice; MPTP/p+ Isoorientin, n = 10 mice) in (B–D) the open field test, E) the rotarod test, and F) the pole test. G,H) Immunohistochemical staining and counting of TH^+^ neurons in the SNpc. n = 10. Scale bar, 100 mm. I,J) Immunohistochemical staining and counting of Nissl‐positive neurons in the SNpc. n = 10. Scale bar, 100 mm. K) The midbrain tissues were collected from the Isoorientin and MPTP/p group, followed by tissue proteins that were immunoprecipitated with anti‐SHMT1 antibody, and then the samples were analyzed by immunoblotting. L–N) The levels of Phosphatidylcholine (PC), SAM, and glutamate (Glu) are detected in the midbrain. n = 6. O,P) Representative co‐localization images of GS/EAAT2 and astrocyte marker GFAP in the MPTP/p mouse model. Scale bar, 20 µm. Q) Immunoblot analysis of GS, EAAT2, H3K4me1, and TH in the midbrain. n = 3. Data were analyzed using two‐way ANOVA, followed by Tukey post‐hoc tests, and presented as mean ± SEM. ^*^ *p* < 0.05, ^**^ *p* < 0.01, ^***^ *p* < 0.001, ns: no significance.

Subsequently, we evaluated the neuroprotective effect of Iso in vivo. As illustrated in **Figure** **7**A, after treatment with Iso, mice underwent the behavioral tests. Results showed significant improvements in motor function during the open field test, with increased distance traveled and higher speed (Figure 7B–D). Latency to fall decreased, and the time taken for T‐turn increased in MPTP/p mice, which was ameliorated by Iso treatment (Figure 7E,F). Subsequently, mice in the Iso group exhibited significantly more TH^+^ neurons and Nissl‐positive neurons compared with the MPTP/p group (Figure 7G–J). As shown in Figure 7K, pretreatment with Iso promoted the combination between SHMT1 and PEMT in the striatum. Levels of PC and Glutamate were decreased, while SAM levels increased in the striatum of Iso‐treated mice (Figure 7L–N). Consistently, immunofluorescence staining indicated that colocalization of EAAT2/GLUL with GFAP was highly increased following Iso treatment (Figure 7O,P). The GLUL, EAAT2, and H3K4me1 levels were upregulated in the striatum in response to Iso pretreatment (Figure 7Q; Figure S12, Supporting Information). These results reveal that Iso facilitates the SHMT1‐PEMT combination, mitigates the glutamate excitotoxicity, and provides neuroprotection in the MPTP/p mouse model, indicating that Iso is a promising lead compound for the treatment of PD.

## 3 Discussion

We report that one‐carbon metabolism is markedly impaired in pathological conditions of PD patients and the PD mouse model. Our results also indicate that knockout of astrocytic SHMT1 impairs one‐carbon metabolism by disrupting methyl donor SAM availability, thereby impacting the interaction with PEMT and modulating EAAT2/GS synthesis to maintain homeostasis in the Glutamate‐Glutamine cycle.

Importantly, we first describe a novel molecular function of SHMT1 in the SAM availability. Previous investigations have shown that metabolic changes affecting SAM availability, such as methionine starvation or disruptions in one‐carbon metabolism.^[^ ^16^ ^]^ Moreover, Glucose‐derived ribose and one‐carbon units, supplied by both glucose and serine metabolism, are synergistically integrated into the methionine cycle through de novo ATP synthesis and fueling the generation of SAM during LPS‐induced inflammation.^[^ ^17^ ^]^ As we know, SAM levels are dynamically regulated by key intracellular enzymes and sensors, including methionine adenosyltransferases (MATs), which catalyze SAM synthesis from methionine and ATP, and SAMTOR, which couples SAM availability to mTORC1 signaling.^[^ ^3^ ^]^ Mechanistically, SAM binding to the methyltransferase (MTase)‐like domain of SAMTOR induces a conformational shift in its N‐terminal helix, obstructing interaction with the GATOR1‐KICSTOR complex and thereby alleviating inhibition of mTORC1 to promote anabolic growth pathways.^[^ ^18^ ^]^ Recently, SAM scarcity was reported to be sensed by receptor‐interacting protein kinase 1 (RIPK1). SAM depletion (via methionine restriction or MAT2A inhibition) reduces this methylation, unleashing RIPK1 self‐assembly, caspase‐8‐dependent apoptosis, and proinflammatory cytokine production (e.g., TNF‐α, IL‐6). This links metabolic stress to inflammation and tissue damage, evidenced by accelerated liver steatosis, fibrosis, and cancer in PRMT5‐deficient or methionine‐restricted mice.^[^ ^19^, ^20^ ^]^ Collectively, the SAM‐sensing network, encompassing RIPK1, SAMTOR, and associated effectors, transduces nutritional cues into cellular fate decisions via allosteric regulation, posttranslational methylation, and metabolite feedback loops, offering novel therapeutic avenues for metabolic disorders, including inflammatory diseases,^[^ ^11^ ^]^ obesity,^[^ ^21^ ^]^ and cancer.^[^ ^22^ ^]^

Furthermore, SAM consumption extends beyond nucleic acid and protein methylation, with PE methylation accounting for over 80% of SAM utilization in certain contexts; PE depletion elevates SAM pools, leading to histone hypermethylation and altered chromatin states.^[^ ^12^ ^]^ SAH, the byproduct of SAM‐dependent reactions, provides critical feedback inhibition by competitively antagonizing methyltransferases, while its hydrolysis by SAH hydrolase (AHCY) sustains the SAM/SAH ratio (methylation index).^[^ ^23^ ^]^ Perturbations in this balance—arising from folate or vitamin B12 deficiencies, AHCY mutations, or metabolic dysregulation—result in SAH accumulation, broadly impairing methylation‐dependent processes such as histone remodeling and G‐protein‐coupled receptor signaling.^[^ ^24^ ^]^ In our study, we observed that SHTM1 upregulation did not influence the biosynthesis of SAM in the PD model. Our data showed that SHMT1 affects the PEMT‐catalyzed demethylation Pathway, which dynamically regulates the PC/PE ratio in membranes, impacting the availability of SAM. Moreover, due to the complexity of Parkinson's disease animal models, the current trend is to use these models in combination to compensate for the shortcomings of a single model.^[^ ^25^ ^]^ Neurotoxin‐induced animal models (such as MPTP/p, 6‐OHDA et.al) remain popular due to their cost‐effectiveness in generating a PD‐like phenotype in a relatively short time span. Using the animal models injected with PFFs or viral vectors encoding SNCA gene copies will be suitable for studying initiation and propagation of α‐syn pathology. In our work, we comprehensively used MPTP/p, PFF, and PFF/a‐syn AAV models to verify the neuroprotective function of SHMT1 in PD. In conclusion, this study addresses a critical gap in understanding PD pathogenesis by identifying a novel astrocyte‐specific epigenetic‐metabolic axis.

Protein–protein interactions (PPIs) are fundamental to many biological processes and play an important role in the occurrence and development of a variety of diseases. Targeting the interactions between PD‐related proteins with emerging small‐molecule drugs has become an attractive approach for the treatment of PD. Therefore, small molecules can act as molecular gels to promote binding by inducing PPI.^[^ ^26^ ^]^ There are different types of PPI modulators. Our previous study revealed that talnifumate, as a negative modulator, impedes the combination of NLRP3 and ASCT2.^[^ ^27^ ^]^ Iso is a kind of natural C‐glucosyl flavone extracted from Pueraria lobata tubers. It showed multiple pharmacological activities on different diseases, such as diabetes and obesity.^[^ ^28^ ^]^ In our system, we found that it can boost the combination of SHMT1 and PEMT, thereby promoting the production of SAM, which leads to increased expression of EAAT2 and GS, contributing to the neuroprotective effect in PD. However, based on the current experimental results, we cannot conclude that the combination of SHMT1/PEMT is a direct binding. In addition to the methods we use, other approaches, including the yeast two‐hybrid system, pull‐down assays, affinity purification‐mass spectrometry (AP‐MS), and bimolecular fluorescence complementation (BiFC), can be further applied in protein‐protein interactions.^[^ ^29^ ^]^

Given the pivotal role of the SHMT1/PEMT axis in PD, we believed that targeting this axis could represent a novel intervention strategy for PD. Our work not only elucidates the novel molecular mechanisms underlying SAM availability but also demonstrates the enhancement of H3K4me1 methylation and upregulation of EAAT2/GS expression. Moreover, Small‐molecular Isoorientin, which was screened from the natural product library, enhances the interaction between SHMT1 and PEMT can be a promising strategy for PD treatment.

## 4 Experimental Section

### Nontargeted Metabolomics for the Patient Sample

*Sample Preparation*: Panomix Biotech Inc. (Suzhou, China) provided the non‐targeted metabolomics service using previously published procedures.^[^ ^30^ ^]^ Peripheral blood samples were collected from patients with Parkinson's disease and healthy controls. The characteristics of the healthy control and PD patient groups are detailed in Table S1 (Supporting Information). Briefly, the blood samples were stored in the EDTA‐pretreated tube and centrifuged at 1000 rpm for 5 min. The uppermost plasma layer was carefully collected and placed in a new RNase‐free tube. Plasma samples were then analyzed according to the manufacturer's instructions.

This study was performed in line with the principles of the Declaration of Helsinki. Approval was granted by the Ethics Committee of the Affiliated Brain Hospital of Nanjing Medical University (Approval No: 2020‐KY043‐01, Nanjing, China).

*Data pre‐processing*: The raw data obtained were converted to mz XML format (xcms input file format) using Proteowizard software (v3.0.8789). Peak identification, filtration, and alignment were performed using the XCMS package in R (v3.3.2). Subsequently, data, including mass to charge ratio (m/z), retention time, and peak area (intensity) were obtained. In positive ion mode, 4449 precursor molecules were identified, while in negative ion mode, 8592 precursor molecules were obtained. The data were exported to Excel for further analysis. To facilitate comparisons across different levels, the data were normalized based on peak area.

*Bioinformatics analysis*: MetPA, part of MetaboAnalyst (www.metaboanalyst.ca), is mainly based on the KEGG metabolic pathway framework. The MetPA database identifies possible bioturbated metabolic pathways through metabolic pathway enrichment and topology analysis. The metabolic pathways of metabolites were analyzed and associated with differential metabolites by the MetPA database. Meanwhile, the data analysis was done by the hypergeometric test and applied pathway topology with the Relative‐betweenness Centrality.

### Mice

C57BL/6 mice (6–8 weeks) were housed under specific pathogen‐free (SPF) conditions. All animal experiments were approved by the Animal Protection and Ethics Committee of Nanjing Medical University (IACUC No. 2 008 067).

### The MPTP/p Mouse Model

Mice were randomly assigned to receive either MPTP/p (MPTP: 20 mg kg^−1^, s.c., and probenecid: 250 mg kg^−1^, i.p.) or saline twice weekly for five weeks. One week after the final injection, mice were euthanized, and samples were collected for subsequent analyses.

### The α‐Synuclein PFF Mouse Model

*Pretreatment* of *a‐Syn PFF*: Recombinant Human Alpha‐synuclein protein aggregates (Abcam, ab218819) at a concentration of 2 mg ml^−1^ were sonicated before use with parameters set to 30% power, 01s on, and 01s off for 10s, repeated 6 times. Validation was conducted using the electron microscope (Figure S6B, Supporting Information).

*Preparation of PFF mouse model injected in the striatum*: A total modeling duration of 9 months was employed. Commercialized PFF protein (5 µg/side) was unilaterally injected into the striatum to validate efficiency (Figure S6C–E, Supporting Information). Mice were maintained and monitored for motor performance at 3, 6, and 9 months. Immunohistochemical staining is shown in Figure S6F (Supporting Information).

*Preparation of PFF mouse model injected in the midbrain*: Following previously reported,^[^ ^13^ ^]^ a total modeling duration of 3 months was employed. LV‐mSHMT1 and α‐Syn overexpression AAV were mixed (1:1) and bilaterally injected into the SNpc region. After being expressed for 4 weeks, mice were injected with PFF (5 µg per side), followed by behavioral testing 2 months later.

### Stereotaxic Surgery

The HBLV‐m‐*Shmt1*‐3xFLAG‐ZsGreen‐PURO and its overexpression control HBLV‐ZsGreen‐PURO were purchased from Hanheng Bio (Shanghai, China). Mice were anesthetized using isoflurane via an RWD gas anesthesia system and secured in a stereotaxic apparatus (RWD Life Science, Shenzhen, China). The animals were anesthetized and microinjected in the substantia nigra pars compacta (SNpc) (A/P −3.0 mm, R/L ±1.3 mm, D/V −4.5 mm).

### Nontargeted Metabolomics for the PFF Mice Model Sample

*Sample Preparation*: After the PFF mouse models were injected into the midbrain, both the midbrain and striatum were collected for Metabolomics analysis. Polar metabolites were extracted from approximately the tissues using 500 µL of ice‐cold methanol: H_2_O (4:1, v/v) containing 0.224 mm phenylhydrazine. Tissues were minced into small pieces in the extraction solvent, then homogenized on a bead ruptor using an optimized programme. Tissue homogenates were incubated at 1500 rpm for 30 min at 4 °C. Following the incubation, samples were kept at −20 °C for 1 h for the derivatisation of alpha‐keto acids. Then, samples were centrifuged for 10 min at 12 000 rpm at 4^ o^C. Clean supernatant was transferred to a new tube. Extraction was repeated for another round by adding another 500 µL of ice‐cold methanol containing 0.224 mm phenylhydrazine. Extracts from both rounds were pooled into a single tube and dried in a SpeedVac under H_2_O mode. The dried extract was reconstituted in 5% acetonitrile in water before LC‐MS analysis on an Agilent 1290 II UPLC coupled to Sciex 5600+ quadrupole‐TOF MS.For reverse phase liquid chromatography (RPLC), polar metabolites were separated on a Waters ACQUITY HSS‐T3 column (3.0 × 100 mm, 1.8 µm). MS parameters for detection were: ESI source voltage positive ion mode 5.5 kV; vaporizer temperature, 500 °C; drying gas (N2) pressure, 50 psi; nebulizer gas (N2) pressure, 50 psi; curtain gas (N2) pressure, 35 psi. The scan ranges were set at m/z 60‐700 during RPLC. Information‐dependent acquisition mode was used for MS/MS analyses of the metabolites. Collision energy was set at (+) 35 ± 15 eV.

*Data acquisition and processing*: It was performed using Analyst TF 1.7.1 Software (AB Sciex, Concord, ON, Canada). All detected ions were extracted using MarkerView 1.3 (AB Sciex, Concord, ON, Canada) into Excel in the format of a 2D matrix, including mass to charge ratio (m/z), retention time, and peak areas, and isotopic peaks were filtered. PeakView 2.2 (AB Sciex, Concord, ON, Canada) was applied to extract MS/MS data and perform comparisons with the Metabolites database (AB Sciex, Concord, ON, Canada), HMDB, and standard references to annotate ion identities. Peak areas of endogenous metabolites were normalized to the areas of their corresponding isotopically labeled structural analogues for quantitation. For endogenous metabolites without labeled structural analogues, an automated algorithm selects the optimal internal standard for quantitation based on the rule of minimal coefficients of variation (COVs) after normalization.

### SAM Detection Administration

*SAM detection*: SAM levels were detected using an ELISA Kit (Biorbyt, Cat#orb781945; Shanghai), following the manufacturer's protocol.

*SAM detection administration*: After the preparation of the PFF mouse model injected in the striatum, animals were anesthetized and microinjected into the lateral ventricular region of the brain (AP: −0.35 mm, ML: ±0.7 mm, DV: −2.4 mm). For SAM administration, it was aspirated through the inner injection tube using a syringe pump. The microinjection was performed at a dose of 100 ng/side in 0.4 µL over 15 min. Following the injection, the catheter was left in place for ≈10 min. Behavioral tests were conducted 7 days after the administration of SAM.

### Behavioral Test

*Open field test*: The experiment utilized a cubic behavioral testing chamber (50 cm × 50 cm × 50 cm, L×W×H). Before formal testing, all mice underwent a 10‐min habituation session in the chamber. After each habituation phase, the chamber walls and floor were thoroughly cleaned with 75% ethanol to eliminate odor cues and fecal residues. During the formal testing phase, mice were placed at the center of the chamber, and their movement trajectories were continuously recorded for 5 min using the TopScan Animal Behavior Analysis System (TopScan Realtime Option v2.00, Clever Sys Inc., VA, USA) to quantitatively assess spontaneous locomotor activity.

*Pole test*: The experimental setup consisted of a vertical wooden pole (50 cm in height). Before formal testing, mice underwent habituation sessions, during which each animal was placed atop the pole to spontaneously descend, with three daily trials conducted to minimize environmental stress. During the formal experiment, two parameters were quantified: total descent duration (time from the top to base of the pole) and reversal duration (time spent on directional changes during descent).

*Rotarod test*: Before formal testing, all mice underwent acclimation training on a rotarod apparatus, consisting of three consecutive trials at a constant speed (5 rpm), each lasting 3 min with inter‐trial intervals of at least 15 min. The formal test employed a linear acceleration protocol (starting at 5 rpm, peaking at 25 rpm, with a 1‐min acceleration period) during a 3‐min session. Motor coordination was assessed by recording fall latency.

*Gait analysis*: Mice underwent a 7‐day habituation period in a 50×6 cm corridor with access to shelter to reduce stress. Locomotor function was evaluated using an automated gait analysis system (CatWalk XT) on a reflective glass walkway illuminated by green LED light. Paw dynamics and gait trajectories were quantified from high‐speed video recordings (100 fps) during three consecutive trials per mouse.

### ELISA Assays

PEMT and PC levels were detected using the ELISA Kit (Jianglai Biotechnology Co., Ltd, Cat#JL51290‐96T; Elabscience, Cat#E‐BC‐K796‐M). Experimental procedures were performed following the manufacturer's protocol.

### Glutamate Detection

Astrocytes and midbrain tissues were homogenized, and the supernatants were analyzed using a detection kit (Solarbio, Cat#BC1585) according to the manufacturer's protocol.

### Primary Cell and Cell Line Cultures

*Primary astrocyte isolation*: Postnatal C57BL/6 pups (24‐72 h) were sterilized with 75% ethanol and euthanized by cervical dislocation. Brains were dissected along the sagittal suture using sterile tools, washed twice in ice‐cold high‐glucose DMEM (containing 100 U mL^−1^ penicillin‐streptomycin), and cortical tissues were isolated under the stereomicroscope (40×). Tissue fragments (1 mm^3^) underwent the trypsin‐EDTA digestion (0.25%, 37 °C/5% CO_2_), which was terminated by adding 10% FBS‐DMEM. After filtration and centrifugation (1000×g, 5 min), cells were resuspended in complete medium (DMEM, 10% FBS, 1% GlutaMAX) and plated on PLL‐coated flasks (0.1 mg mL^−1^). The medium was refreshed at 12 h (to remove debris) and every 72 h.

*HEK‐293T cell line (RRID: CVCL_0063*): The cell line was purchased from the Cell Bank of the Chinese Academy of Sciences (Cat#GNHu17, Shanghai, China), where it was characterized by mycoplasma detection and STR identification (Amelogenin: X; D5S818: 8,9; D13S317: 12,14; D7S820: 11; D16S539: 9,13; vWA: 16,19; TH01: 7,9.3; TPOX: 11; CSF1PO: 11,12; D19S433: 18; D21S11: 28,30.2; D18S51: 17,18). The cell was cultured at 37 °C and 5% CO_2_, and passaged when it reached 80% confluency. Cells were rinsed with PBS, dissociated using 0.25% trypsin‐EDTA (Gibco) for 2 min at 37 °C, and neutralized with DMEM containing 10% FBS and 1% penicillin‐streptomycin. After centrifugation (1000 × g, 5 min), the cells were resuspended in fresh medium and seeded at the desired density. Experiments commenced when cultures reached 70–80% confluency.

### Cell Viability and LDH Release Assays

Cell viability was assessed using the CCK‐8 assay (Solarbio, CA1210) following the manufacturer's protocols. Briefly, 10 µL CCK‐8 reagent was added to each well of a 96‐well plate, incubated for 2 h at 37 °C, and absorbance was measured at 450 nm (Bio‐Rad 680 microplate reader).

For LDH release quantification, supernatants were mixed with LDH detection reagent (Solarbio, BC0680), incubated for 30 min at RT, and absorbance read at 490 nm. Data were normalized to untreated controls (viability) or lysed cells (cytotoxicity). All experiments included blank corrections.

### QT‐PCR

Total RNA was isolated from homogenized mouse midbrain, striatum, and primary astrocytes using TRIzol reagent (Thermo Fisher, #15596018CN) and quantified via a OneDrop spectrophotometer. Reverse transcription was performed using the HiScript III kit (Vazyme, #R323‐01) per the manufacturer's guidelines. QT‐PCR reactions were conducted with ChamQ SYBR Green Master Mix (Vazyme, #Q341‐02). The primers are listed in Table S2 (Supporting Information).

### Western Blotting

*Histone extraction from cells and tissues*: Following the manufacturer's instructions, the total histones were isolated from cells and tissues using the EpiQuik Total Histone Extraction Kit (Epigentek, Cat# OP‐0006). Cell and tissue lysis samples were prepared as previously reported.^[^ ^31^ ^]^

*Cell protein sample preparation*: Following treatment, cells were rinsed with ice‐cold PBS to remove residual medium. RIPA lysis buffer containing protease inhibitors (1×PMSF) was added to each well, and cells were lysed on ice for 15 min. Lysates were scraped into 1.5 mL microcentrifuge tubes and centrifuged at 16 000 × g (4 °C, 15 min). Supernatants were collected for protein quantification via the BCA assay (BSA standard curve), mixed with 5× SDS loading buffer (1:4 ratio), and heat‐denatured at 95 °C in a metal bath for 5 min. The samples were then aliquoted and stored at −80 °C.

*Tissue protein sample preparation*: Fresh or −80 °C‐stored tissues were homogenized (1:20 w/v) in RIPA buffer (Solarbio, AP02L084) with 1× PMSF using LN2‐cooled tubes. Six homogenization cycles (30 Hz, 30s‐on/30s‐off) achieved complete lysis. Lysates were centrifuged (16 000 × g, 4 °C, 15 min), and supernatants underwent a BCA assay with BSA standards. Samples were mixed with 5× loading buffer (β‐mercaptoethanol, 4:1 v/v), heat‐denatured (95 °C, 5 min), aliquoted, and stored at −80 °C. The antibodies are listed in Table S3 (Supporting Information).

### 13C Labeled Serine Tracing

For ^13^C‐serine labeling, the cells were cultured in MEM (without serine, Basal Media, K431015) supplemented with 5 µCi U‐[^13^C]‐serine (Cambridge Isotope Laboratories, Inc., Cat# CLM‐1396‐1) for 12 h. Subsequently, the cells were washed with cold PBS, and metabolites were extracted using 80% cold methanol. The Shanghai Metabolome Institute (SMI)‐Wuhan conducted UHPLC‐MS analysis, and the abundance of metabolites was quantified relative to the total area of both labeled and unlabeled carboxylic acid analyte/ amino analyte, following established protocols.

### Immunohistochemistry and Immunofluorescence

*Immunohistochemistry*: Brain sections were rinsed three times for 10 min in PBS and treated with 3% H_2_O_2_/PBS (15 min, RT, protected from light) to block endogenous peroxidases. After washing, sections were blocked with 10% donkey serum/0.3% Triton X‐100/PBST (2 h, RT), followed by incubation with primary antibodies (1:500 in PBST) overnight at 4 °C with shaking. Sections were then washed, incubated with HRP‐conjugated secondary antibodies (1:1000, 2 h, RT), and developed with DAB (KGI Biology, #KGP1045‐100; reaction time optimized microscopically). Ethanol‐dehydrated, xylene‐cleared, and neutral resin‐mounted sections were analyzed using stereology (Olympus BX53/Stereo Investigator).

*Immunofluorescence*: Primary astrocytes on coverslips were fixed with 4% PFA (30 min, RT), washed three times for 10 min with PBS, and blocked in 10% donkey serum/0.3% Triton X‐100/PBST (2 h, RT). Primary antibodies (4 °C, overnight) and fluorescent secondaries (Cy3/Alexa Fluor 488, 1:1000, 2 h, RT) were applied sequentially. After DAPI staining and anti‐fade mounting, imaging was performed using a Nikon TE2000‐S or Zeiss LSM800 microscope.

Mice were perfused with 4% PFA (endpoint: liver pallor, limb rigidity). Brains were post‐fixed (24 h, 4 °C), dehydrated in 20%→30% sucrose (3 days each), and sectioned coronally (30 µm). Sections stored in PBS: glycerol (1:1, −20 °C) were blocked and stained identically to cell samples.

### CHIP Sequence Assay

Astrocyte chromatin accessibility was analyzed using the Hyperactive Universal CUT&Tag Assay Kit for Illumina Pro (Vazyme, TD904), following the manufacturer's protocol with minor modifications. Briefly, 10 000–50 000 mouse primary astrocytes were harvested and washed with pre‐chilled Wash Buffer. Cells were then incubated with Concanavalin A‐coated magnetic beads (ConA Beads Pro) to enable cell immobilization. After bead binding, cells were permeabilized using Digitonin and incubated with a primary antibody against the target protein at 4 °C overnight. Following primary antibody incubation, samples were washed and incubated with a secondary antibody at room temperature for 30–60 min. After washing, pA/G‐Tn5 transposase fusion protein (pA/G‐Tnp Pro) was added to the samples and incubated for 1 h to allow targeting of genomic DNA in proximity to the bound antibody complexes. Subsequent tagmentation was initiated by the addition of TTBL buffer and incubation at 37 °C for 1 h. Genomic DNA fragments were extracted using DNA Extract Beads Pro and purified. Tagmented DNA was then subjected to PCR amplification using CAM (CUT&Tag Amplification Mix) and dual indexing primers (TruePrep Index Kit for Illumina, Vazyme). Amplified libraries were purified using VAHTS DNA Clean Beads (Vazyme #N411) and quantified using a Qubit fluorometer (Invitrogen) and quality‐assessed by Agilent Bioanalyzer. Final libraries were quantified by Qubit 3.0 (VAHTS kit) and sequenced on Illumina NovaSeq 600.

### Co‐IP Assay

Following PBS washes, cells were lysed in ice‐cold NP‐40 lysis buffer supplemented with protease inhibitors for 15 min on ice. Lysates were centrifuged at 16 000 × g (4 °C, 15 min), and supernatants were collected for protein quantification. For input samples, 30 µL of lysate was mixed with 1× SDS loading buffer, heat‐denatured at 95 °C (5 min, metal bath), and stored at −20 °C. For immunoprecipitation, 1 mg of total protein was incubated with primary antibodies overnight at 4 °C. Pre‐equilibrated Protein A/G agarose beads (Millipore, #16‐156) were added to the antibody‐protein complexes and incubated for 4 h at 4 °C with rotation. Beads were pelleted by centrifugation (3000 × g, 4 °C, 5 min) and washed three times with ice‐cold lysis buffer. The precipitated complexes were resuspended in 2.5× SDS loading buffer, while wash fractions were pooled and concentrated with an additional 2.5× buffer. All samples were heat‐denatured at 95 °C for 5 min and either immediately used for Western blot or stored at −20 °C.

### Preparation of Tamoxifen‐Induced Astrocytic *Shmt1* Knockout Mice

*Shmt1* ^flox/flox^ mice and *Aldh1l1‐CreERT2* mice were purchased from GemPharmatech (Nanjing, China). Astrocytic‐specific knockout mice were generated by crossing *Aldh1l1*‐CreERT2 mice with *Shmt1* ^flox/flox^ mice. The heterozygous *Aldh1l1‐*Cre/WT/*Shmt*1^flox^/WT mice were consecutively self‐crossed to obtain astrocytic‐specific *Shmt1* CKO mice. The mouse genotype was identified by the One Step Mouse Genotyping Kit (Atgbiotechnology, China). Induction of Cre recombinase activity was achieved using tamoxifen (Sigma, 100 µg/g body weight) dissolved in corn oil (Solarbio, China) and administered intraperitoneally (i.p.) for 5 consecutive days, followed by a 1‐week metabolization period. After tamoxifen administration, astrocytes from CKO mice were identified by immunofluorescence staining to confirm the deletion of *Shmt1*.

### High‐Performance Liquid Chromatography (HPLC)

Mice were sacrificed and the striatum was collected to measure the levels of monoamine transmitters (DA, 5‐HIAA, HVA, and 5‐HT). Samples (10 µL mg^−1^) were homogenized in a buffer containing 0.1 mol L^−1^ perchloric acid, 0.1 mm EDTA‐2Na, and 4 × 10^8 ^mol L^−1^ DHBA (Sigma, 858 781), and then centrifuged at 20 000 g for 20 min at 4 °C. The supernatant was collected for measurement. The HPLC detection system and Parameters were set as previously described.^[^ ^32^ ^]^

### Proximity Ligation Assay (PLA)

Protein interactions in astrocytes were detected using the Duolink PLA assay kit (Sigma‐Aldrich, DUO92101) following the manufacturer's protocol, as previously reported.^[^ ^27^ ^]^ Slides were incubated with rabbit primary SHMT1 antibody (Novus, NBP3‐36705) and mouse primary PEMT antibody (Biorbyt, orb1880990) at 4 °C overnight, followed by incubation with PLA probe solution for 1 h at 37 °C. Finally, cell nuclei were stained with DAPI (Invitrogen, D1306), and the slides were imaged using the confocal laser scanning microscopy platform Leica TCS SP8.

### Virtual Screening Analysis

The Alphafold 2 database model was applied to predict the potential binding region of the SHMT1 (green, PDB: 8R7H) and PEMT protein (blue, homology modeling). Virtual docking simulations were performed between the binding region and 5088 natural compounds using PyMOL software. Compounds were ranked based on Gibbs free energy scores.

### Cellular Thermal Shift Assay (CETSA)

To detect the thermal stability of SHMT1/PEMT, astrocytes were treated with DMSO (0.1%) and Isoorientin (10 µm) before MPP^+^ treatment for 24 h. Then, IP samples were collected as described in the Co‐IP assay. The respective lysates were divided into smaller (50 µL) aliquots and individually heated at different temperatures for 3 min (Veriti thermal cycler, Applied Biosystems/Life Technologies), followed by cooling for 3 min at room temperature. The appropriate temperatures were set as 37, 47, 57, 67 °C. The heat lysates were centrifuged at 20 000 g for 20 min at 4 °C. The supernatants were transferred to fresh tubes before SDS‐PAGE and Western blot analysis.

### Drug Affinity Responsive Target Stability (DATRS)

To detect the thermal stability of SHMT1/PEMT, astrocytes were treated with DMSO (0.1%) and Isoorientin (10 µm) before MPP+ treatment for 24 h. Then, IP samples were collected as described in the Co‐IP assay, and cell pellets were lysed in NP‐40 buffer. The lysates were incubated on ice for 10 min and centrifuged at 12 000 × g for 10 min at 4 °C. The protein concentration of the supernatant was measured by BCA assay and adjusted to 3 µg/µL with NP‐40. Aliquots (500 µg) were treated with either 10 µm Iso or ddH_2_O, incubated on ice for 1 h and at room temperature for 50 min. Each aliquot was then digested with protease K (1:400 to 1:3200) for 30 min at room temperature. The reaction was stopped by adding 5X loading buffer and heating at 70 °C for 10 min. Samples were analyzed by SDS‐PAGE.

### Statistical Analysis

Data distribution was evaluated for normality (Shapiro–Wilk test) and variance homogeneity (Levene's test); log transformation was applied where required, and potential outliers were assessed using the ROUT method (Q = 1%). Results are presented as mean ± SEM, with exact sample size (n, biological replicates) indicated in each figure legend. Comparisons between two groups were assessed by a two‐sided unpaired Student's t‐test (Welch's correction applied if variances were unequal). For analyses involving three or more groups, one‐way ANOVA followed by Tukey's post hoc test was used, whereas two‐way ANOVA was employed to evaluate main and interaction effects. Statistical significance was defined as α = 0.05, with exact p‐values reported in the figures. All statistical analyses were performed using GraphPad Prism version 9.0.0 (GraphPad Software, San Diego, CA).

## Conflict of Interest

The authors declare no conflict of interest.

## Author Contributions

Y.‐H.C. and R.‐X.Z. contributed equally to this work. Y.H.C. and R.X.Z. performed the majority of the experimental work. N.X.Z., T.T.S., Z.W.Z., Y.J.Z., and B.Y.H. assisted with the experiments. H.Y., R.H.D., L.C., and Y.C. supplied study‐specific materials and relevant details. W.B.Z. and W.G.L. provided the plasma sample of PD patients and analyzed the data. C.W. provided technical support. YL offered insights and wrote the manuscript. YL analyzed the data and prepared the figures. Y.W., G.H., and M.L. conceived and supervised the study and revised the manuscript. All authors reviewed and provided input to improve the manuscript.

## Supporting information



Supporting Information

Supporting Information

## Data Availability

The data that support the findings of this study are available from the corresponding author upon reasonable request.
