# Supplementary material for: Alternative Pathway for Methyl Supply through the Coupling of SHMT1 and PEMT to Maintain Astrocytic Homeostasis in Parkinson's Disease
Source: Adv Sci (Weinh). 2025 Nov 20;13(7):e16794. doi: 10.1002/advs.202516794 (PMC12866788; doi:10.1002/advs.202516794)
Supplement: Supplementary file 1 — Supporting Information [file ADVS-13-e16794-s002.docx]

Supporting Information

Alternative Pathway for Methyl Supply through the Coupling of SHMT1 and PEMT to Maintain Astrocytic Homeostasis in Parkinson's Disease

*Yue-Han Chen^a,1^, Rong-Xin Zhu ^a,e,1^, Nuo-Xi Zhang^b^, Ting-Ting Sun^a^, Xi-Wei Zhang^a^, Yu-Jie Zhao^a^, Ben-Yu He^a^, Hang Yao^a,c^, Ren-Hong Du^a^, Lei Cao^a,c^, Wen-Bin Zhang^d^, Wei-Guo Liu^d^, Yun Cai^b^, Cong Wang^a^, Gang Hu^a,b^, Yao Wei^b^, Yang Liu ^b,f*^, Ming Lu^a,c,e,f*^*

**Supplementary figures**


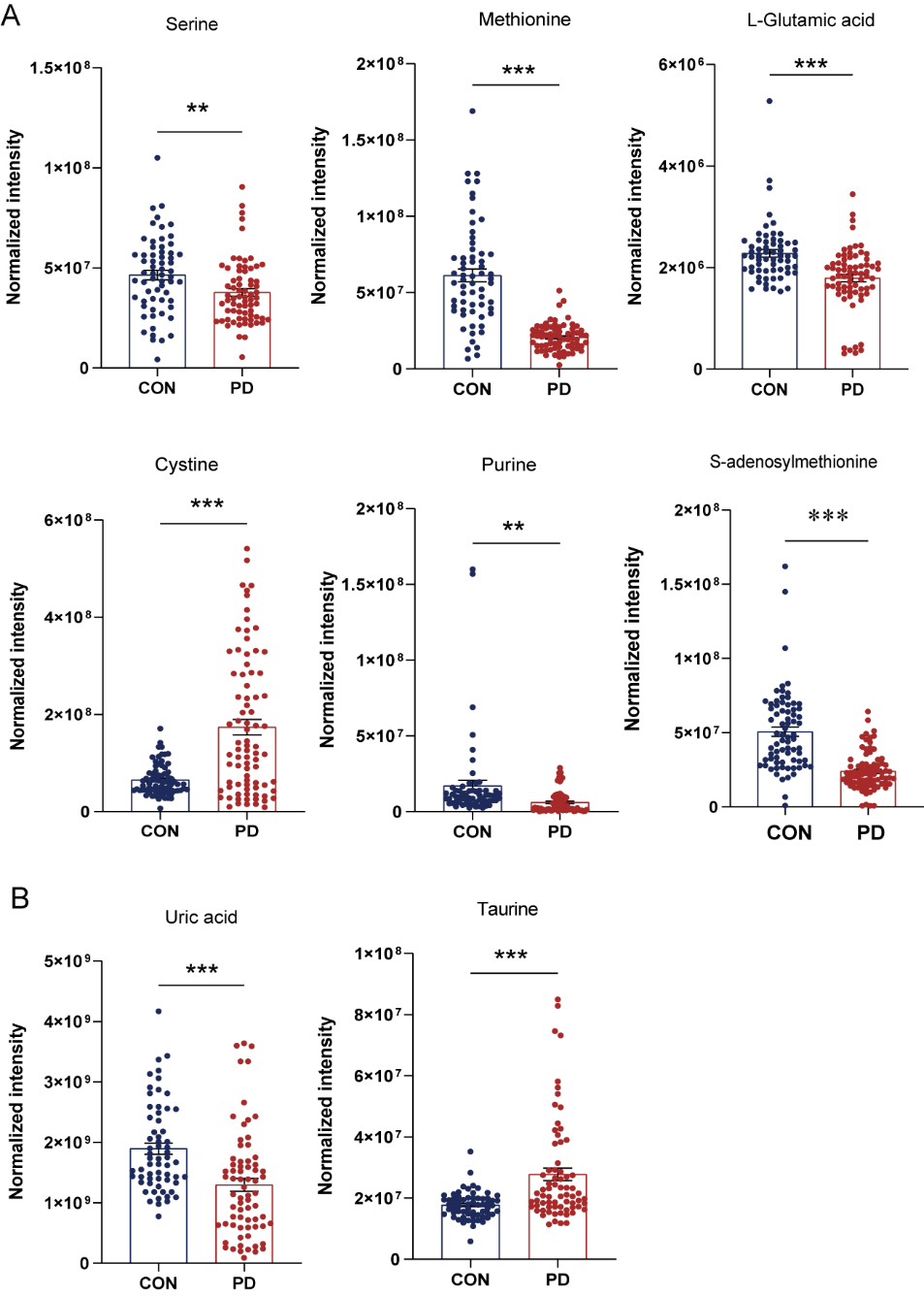


**Figure S1. Metabolomics is used to detect the levels of differential metabolites in the serum of PD patients. (A)** Metabolites enriched in the cysteine and methionine pathways are analyzed. **(B)** Specific PD metabolites, such as taurine and uric acid, were consistent with previous reports from PD patients and healthy controls (PD: n = 70; Control: n = 62). Data were analyzed by an unpaired t-test and presented as mean ± SEM. **P* < 0.05, ***P* < 0.01, ****P* < 0.001, ns: no significance.


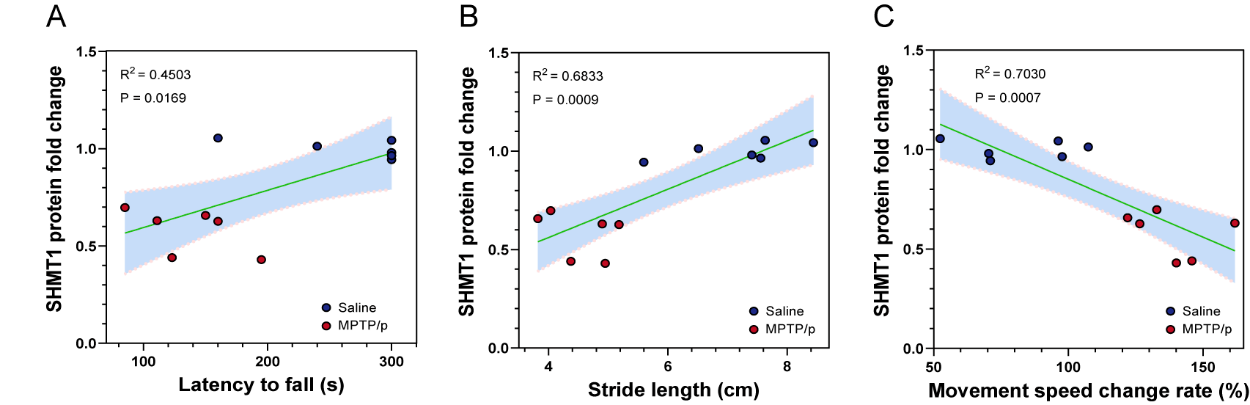


**Figure S2.** The correlation analysis between SHMT1 expression and behavioral test. **(A)** latency to fall in the rotarod test (n=5), **(B)** Stride length in the Elevated plus maze test (n=5), **(C)** Movement speed change rate in the open field test (n=5).


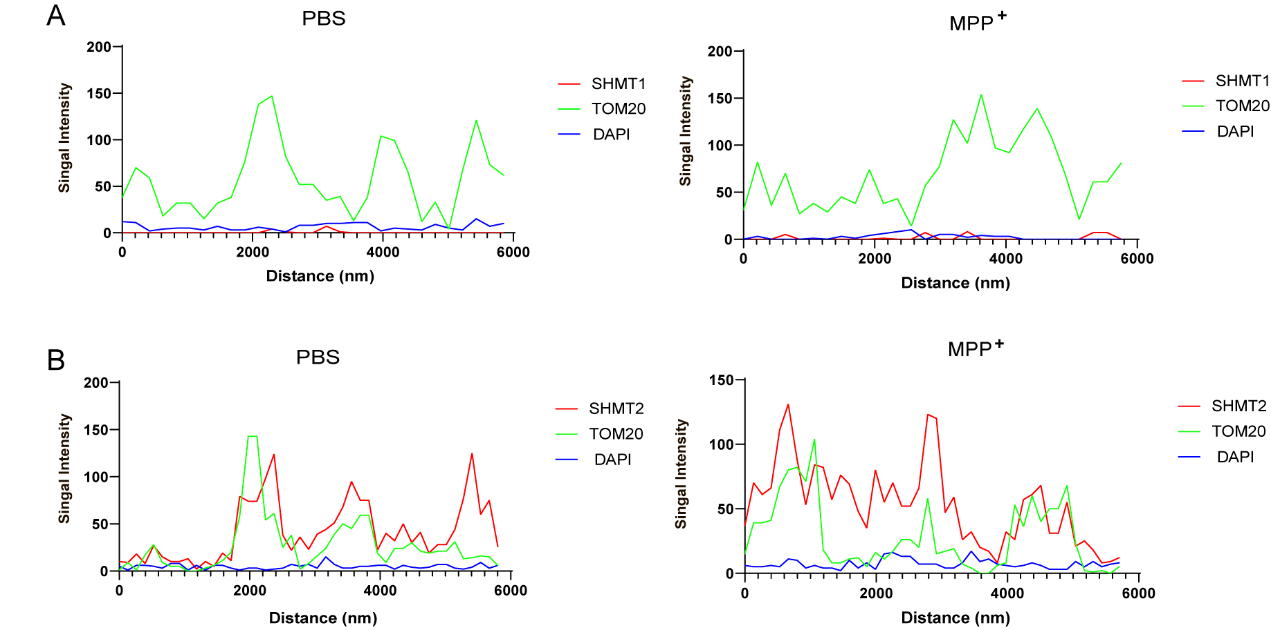


**Figure S3. The distribution of SHMT1 and SHMT2 in the astrocytes. (A)** Co-localization analysis of SHMT1 (red) and Tom 20 (green) in the MPP^+^ model. **(B)** Co-localization analysis of SHMT2 (red) and Tom 20 (green) in the MPP^+^ model.


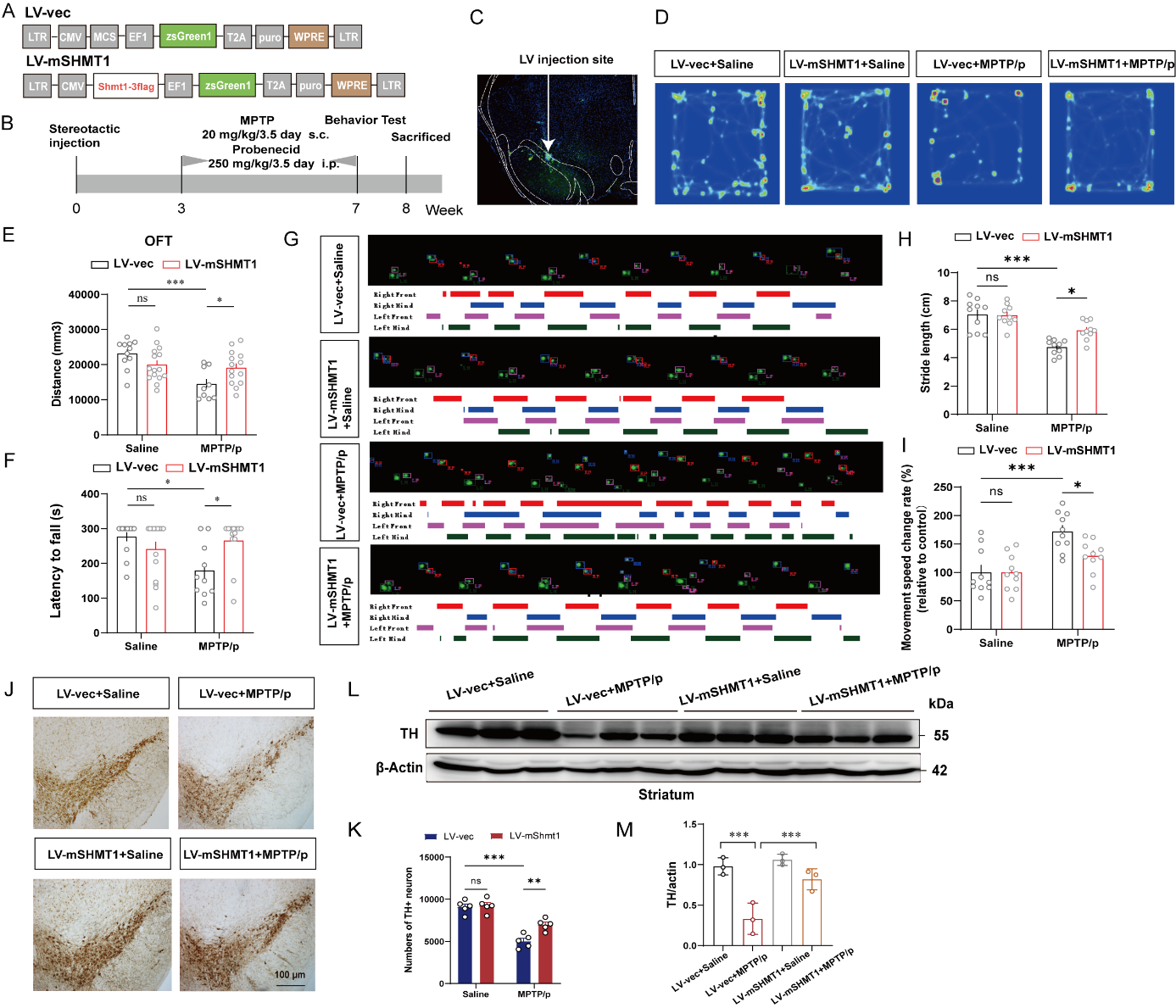


**Figure S4. Overexpression of *Shmt1* alleviates motor symptoms and DA neuronal loss in the MPTP/p mouse model. (A)** Schematic illustration of lentiviral for zsGreen1-vec (Lenti-vec) or zsGreen1-Shmt1 (Lenti-m*Shmt*1). **(B)** Schematic model of the experimental procedure. **(C)** Representative images showing the LV injection site in the SNc region. Motor function performance of mice injected with Lenti-zsGreen1-vec (Lenti-vec, n = 11 mice; Lenti-vec+MPTP/p, n = 9 mice) or Lenti-injected zsGreen1-Shmt1 (Lenti-m*Shmt*1, n = 15 mice; Lenti-m*Shmt*1+MPTP/p, n = 14 mice) in the open field test **(D-E)** and (Lenti-vec, n = 13 mice; Lenti-vec+MPTP/p, n = 10 mice) or Lenti-injected zsGreen1-Shmt1 (Lenti-m*Shmt1*, n = 15 mice; Lenti-m*Shmt1*+MPTP/p, n = 13 mice) in the pole test **(F)**. **(G)** Gait trace of the Lenti-vec and Lenti-m*Shmt1* mice in the Elevated plus maze test. **(H)** Stride length and **(I)** movement speed change rates were analyzed using WalkAnalysisator software (Lenti-vec, n = 10 mice; Lenti-vec+MPTP/p, n = 10 mice) or Lenti-injected zsGreen1-Shmt1 (Lenti-m*Shmt*1, n = 10 mice; Lenti-m*Shmt*1+MPTP/p, n = 10 mice). **(J-K)** Representative immunohistochemical images and quantification of TH-positive neurons in the SN region, n=6. Scale bar, 100 μm. **(L-M)** Representative immunoblot images and quantification of TH in the striatum (n=3). Data were analyzed using two-way ANOVA, followed by Tukey post-hoc tests, and presented as mean ± SEM. **P* < 0.05, ***P* < 0.01, ****P* < 0.001, ns: no significance.


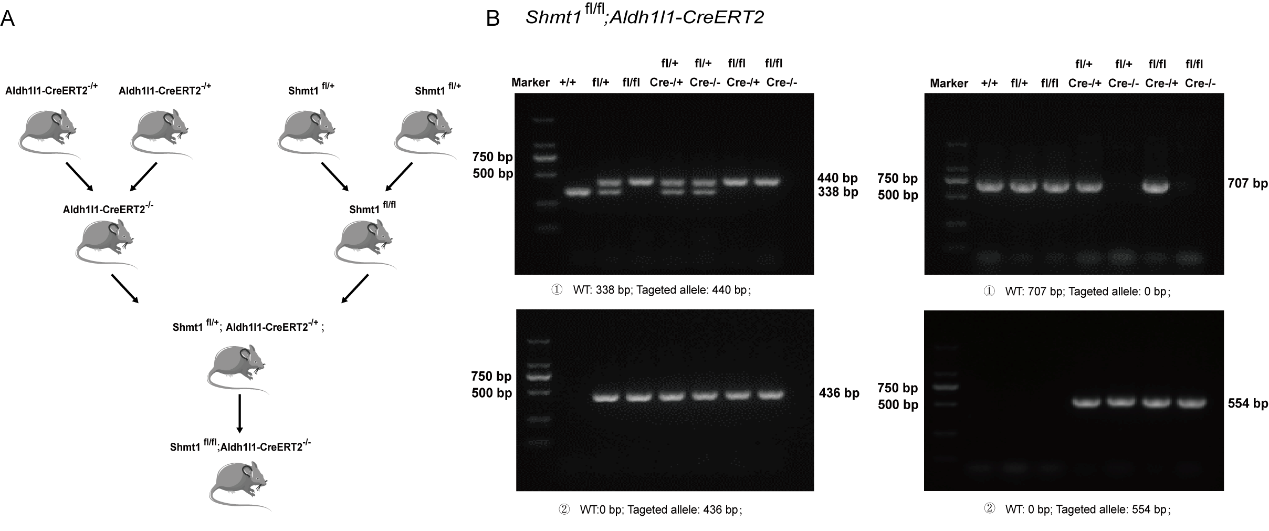


**Figure S5. Construction and validation of Tamoxifen-inducible conditional knockout (CKO) mice *Shmt1^fl/fl^; Aldh1l1-CreERT2*.** **(A)** Construction strategy of the CKO mice *Shmt1^fl/fl^; Aldh1l1-CreERT2.* Panel A schematic was generated in BioRender (license no. *TU28UVIL4Z*) and refined with Adobe Illustrator 2022 (v26.0.0). **(B)** Genotype identification by PCR.


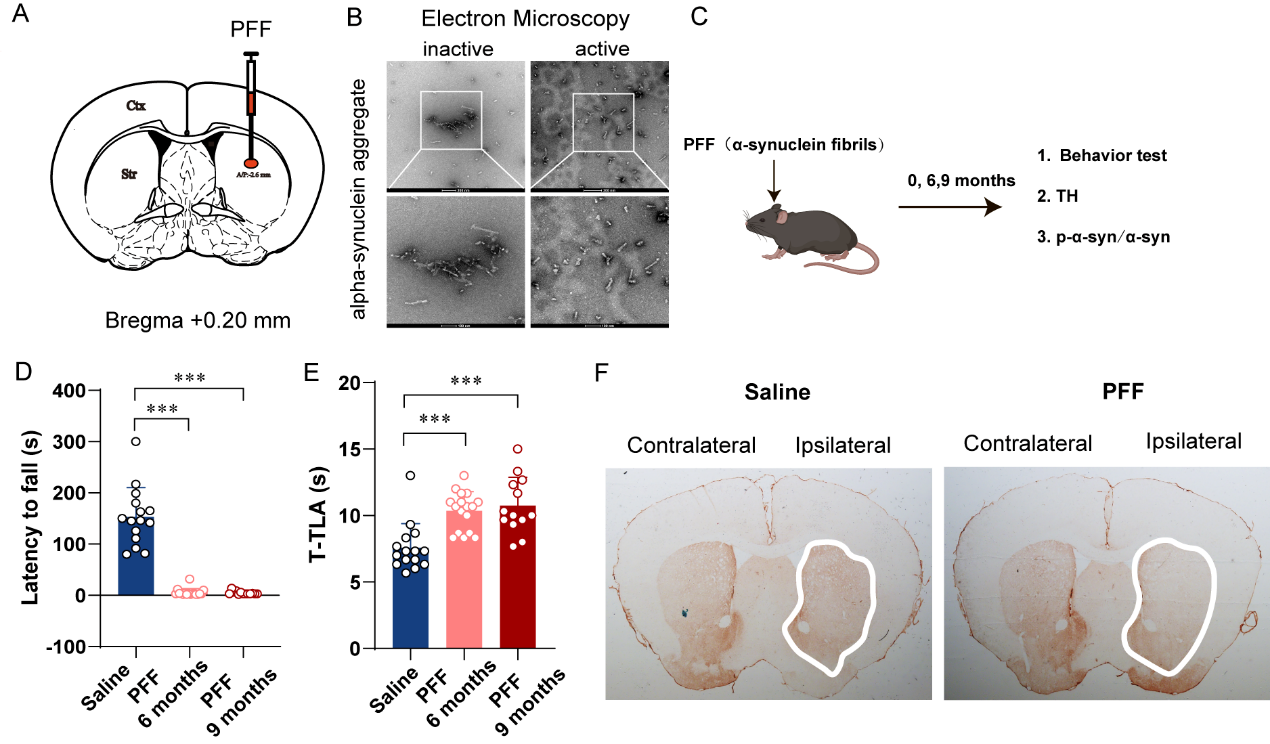


**Figure S6. Construction of the PFF mouse model. (A)** Schematic illustration showing α-synuclein fibrils (PFF) injection site in the striatum. **(B)** Representative images of α-synuclein aggregate in the inactive and active form using electron microscopy. **(C)** Schematic diagram of the experimental procedure. Panel C schematic was generated in BioRender (license no. *TU28UVIL4Z*) and refined with Adobe Illustrator 2022 (v26.0.0). Motor function performance of mice injected with PFF (PBS, n = 15 mice; PFF, n = 18 mice, 6 months; PFF, n = 13 mice, 9 months) in the rotarod test **(D)**, and the pole test **(E)**. **(F)** Representative immunohistochemistry images of TH in the contralateral and ipsilateral regions. Data were analyzed by one-way ANOVA, followed by Tukey post-tests, and presented as mean ± SEM. **P* < 0.05, ***P* < 0.01, ****P* < 0.001, ns: no significance.


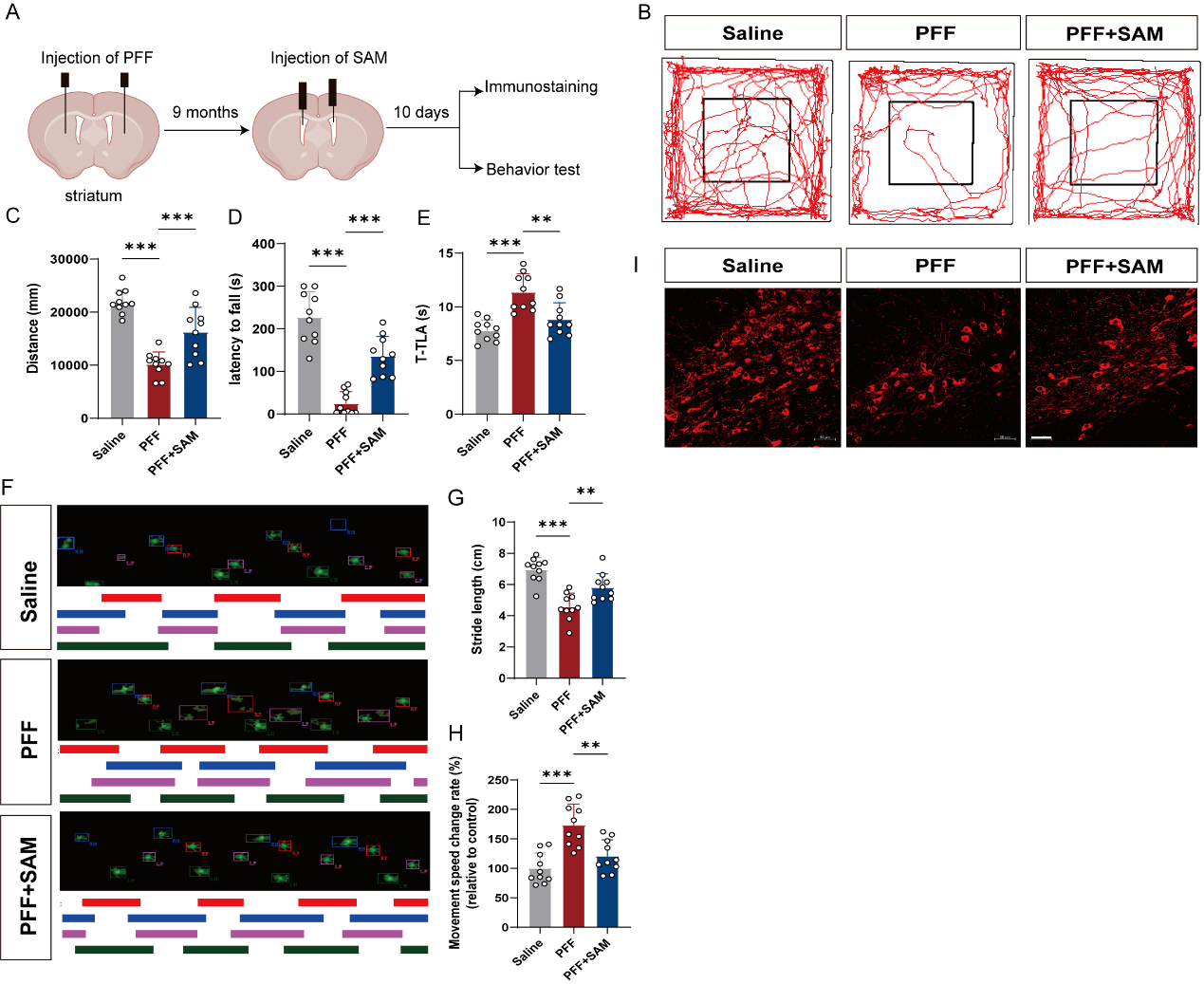


**Figure S7. S-adenosyl methionine (SAM) supplement mitigates the motor dysfunction and the loss of DA neurons in the PFF model. (A)** Schematic diagram of the experimental procedure in the PFF mouse model for SAM supplement. Panel A schematic was generated in BioRender (license no. *TU28UVIL4Z*) and refined with Adobe Illustrator 2022 (v26.0.0). Motor function performance of mice injected with PFF or plus SAM (Saline, n = 10 mice; PFF, n = 10 mice; PFF+SAM, n = 10 mice) in the open field test **(B-C)**, the rotarod test **(D)**, and the pole test **(E)**. **(F)** Gait trace of the Lenti-vec and Lenti-m*Shmt1* mice in the Elevated plus maze test. **(G)** Stride length and **(H)** movement speed change rates were analyzed by WalkAnalysisator software (Saline, n = 10 mice; PFF, n = 10 mice; PFF+SAM, n = 10 mice). **(I)** Representative immunofluorescence images of TH-positive neurons in the SN region. Scale bar, 50 μm. Data were analyzed using one-way ANOVA, followed by Tukey post-hoc tests, and presented as mean ± SEM. **P* < 0.05, ***P* < 0.01, ****P* < 0.001, ns: no significance.


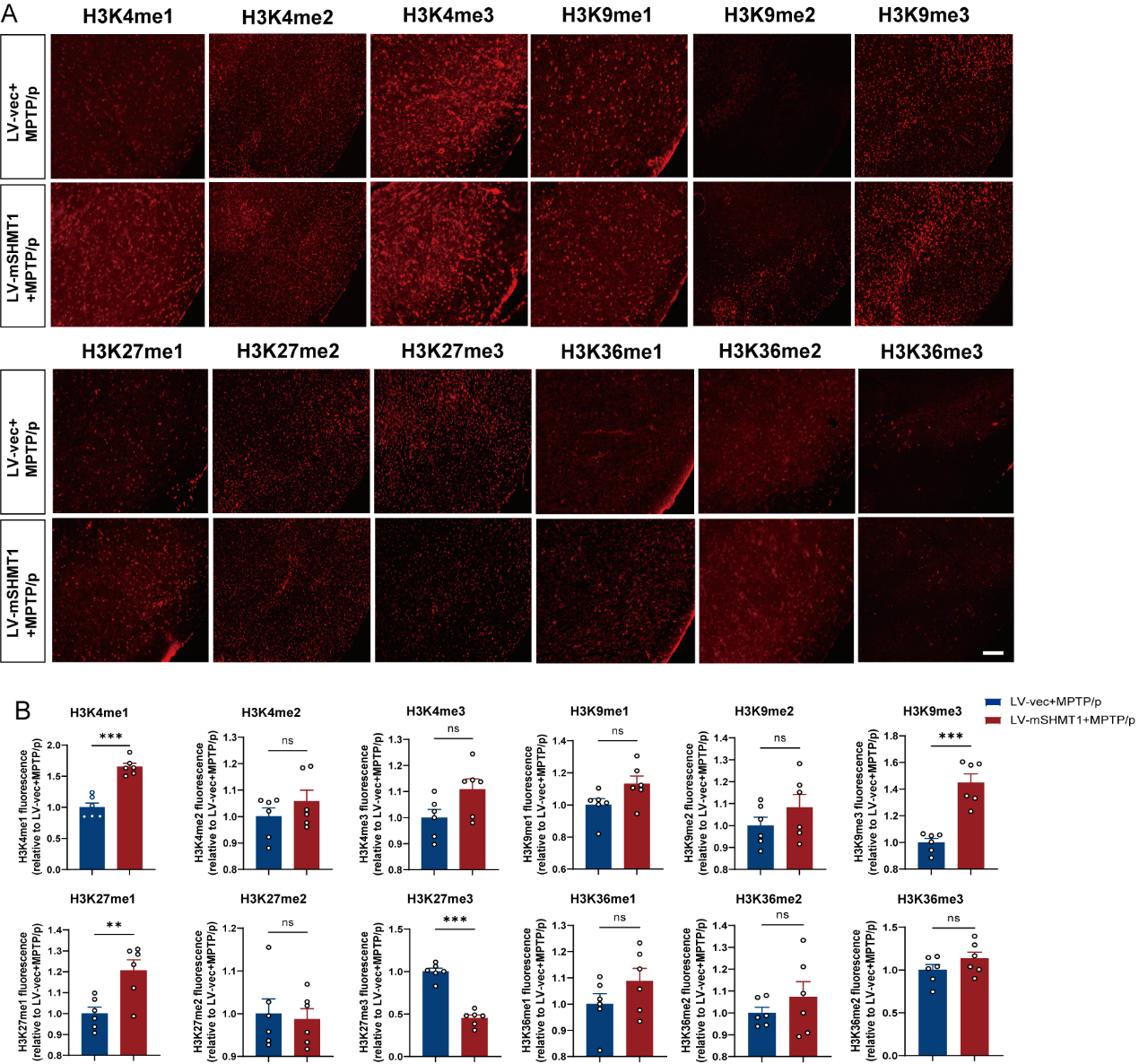


**Figure S8.** **SHMT1 promotes histone methylation *in vivo*. (A-B)** Representative immunofluorescence images and quantification of H3K4me1/2/3, H3K9me1/2/3, H3K27me1/2/3, and H3K36me1/2/3 in the midbrain (n=6). Data were analyzed by an unpaired t-test and presented as mean ± SEM. **P* < 0.05, ***P* < 0.01, ****P* < 0.001, ns: no significance.


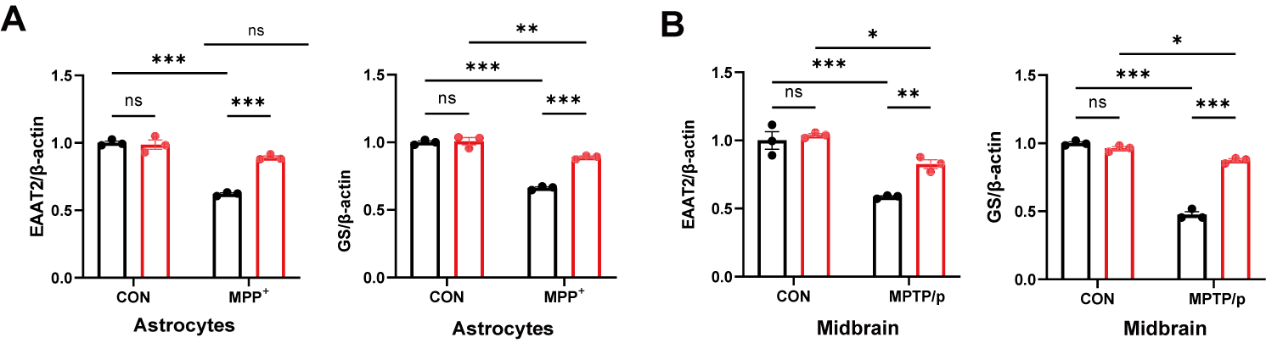


**Figure S9.** Quantification analysis of the immunoblotting of EAAT2 (**A**) and GS (**B**) in astrocytes and striatum. n=3. Data were analyzed by two-way ANOVA, followed by Tukey post-tests, and presented as mean ± SEM. **P* < 0.05, ***P* < 0.01, ****P* < 0.001, ns: no significance.


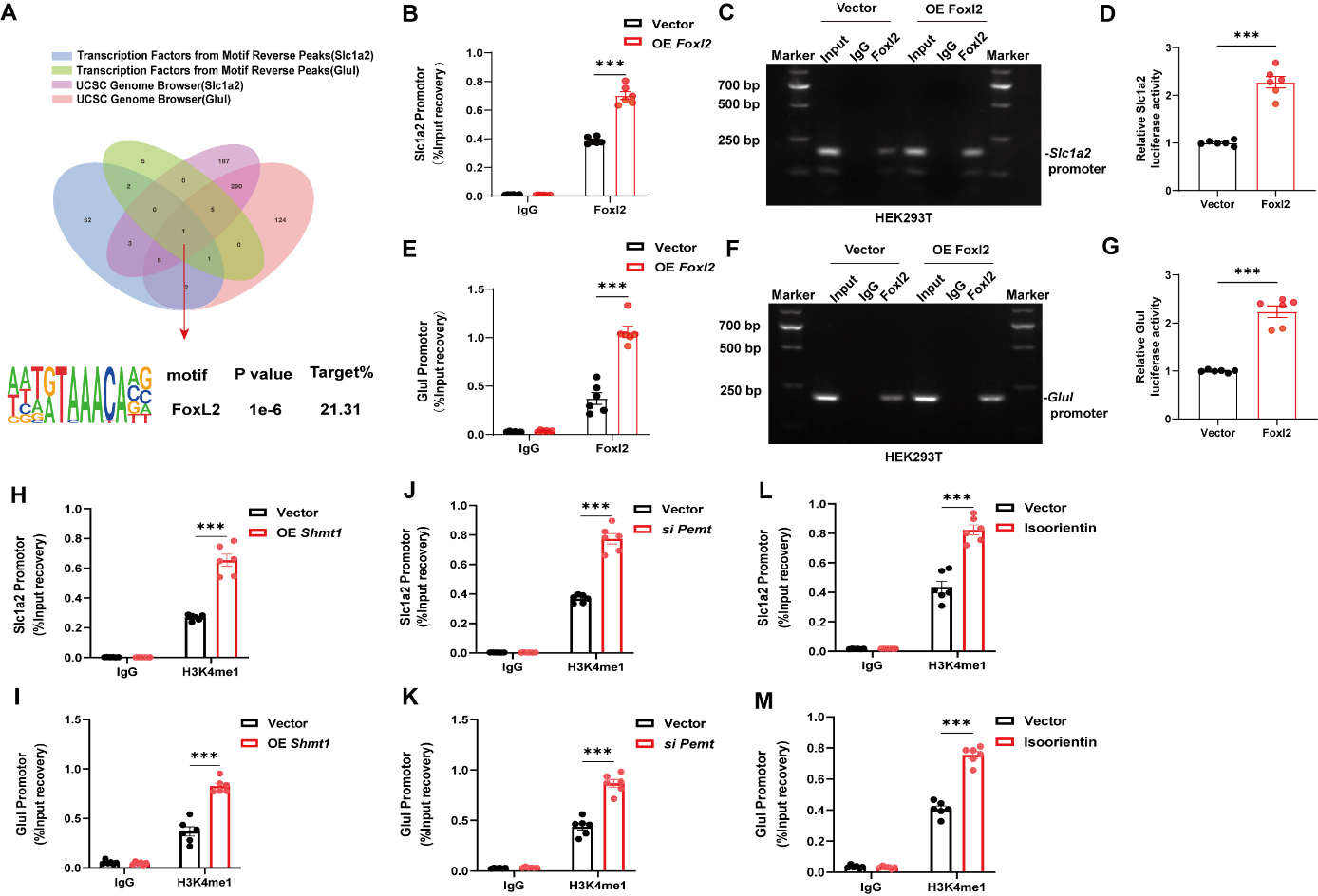


**Figure S10. Foxl2 is a transcription factor for the H3K4me1 enhancer involved in neuroexcitotoxicity. (A)** The Venn diagram showed the potential transcription factor Foxl2. The binding of Foxl2 to the EAAT2 **(B-D)** and GS **(E-G)** promoters was analyzed using ChIP-qPCR in HEK293T cells (three independent experiments). IgG, immunoglobulin G. H3K4me1 ChIP-qPCR on the EAAT2 and GS promoters under SHMT1 overexpression (H-I), PEMT knockdown (J-K), or Isoorientin treatment (L-M). **(B, E, H-M)** Data were analyzed by two-way ANOVA, followed by Tukey post-tests. **(C, F)** Data were analyzed by the unpaired t-test and presented as mean ± SEM. **P* < 0.05, ***P* < 0.01, ****P* < 0.001, ns: no significance.


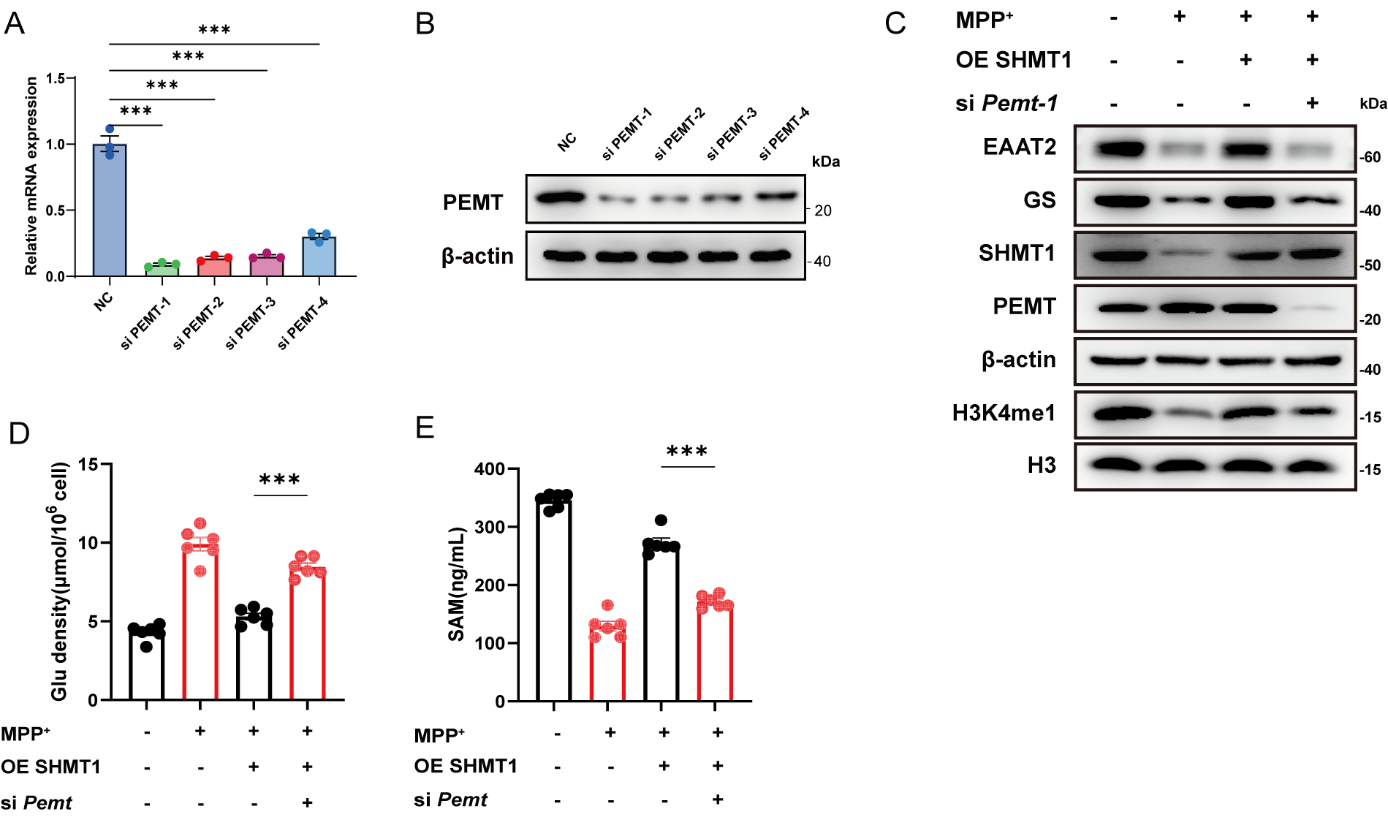


**Figure S11. PEMT deficiency partially reversed the neuroprotective benefit conferred by SHMT1 overexpression against excitotoxicity.** (**A-B**) QRT-PCR and WB validated the efficiency of PEMT knockdown in astrocytes. (**C**) Representative immunoblot images of H3K4me1 and EAAT2/GS expression in astrocytes. (**D-E**) The concentrations of SAM and Glutamate were detected in the PEMT knockdown astrocytes, while SHMT1 was overexpressed. N=3. Data were analyzed by an unpaired t-test. ****P* < 0.001, ns: no significance.


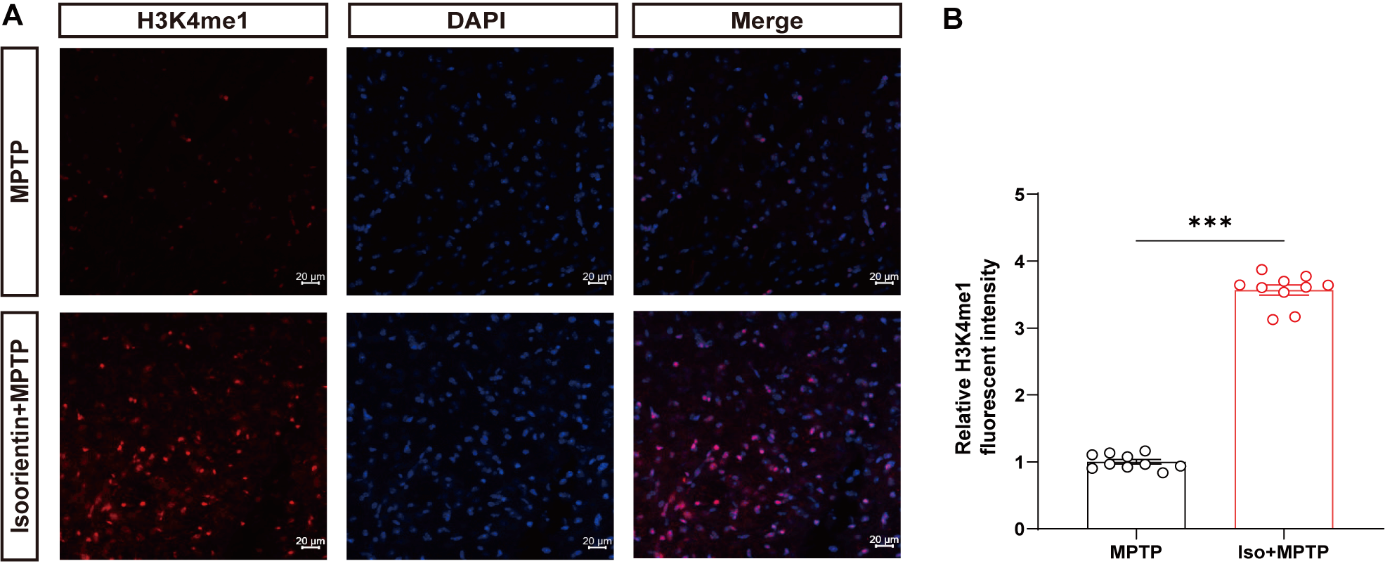


**Figure S12. Isoorientin promotes the H3K4me1 level in the PD model. (A-B)** Representative immunofluorescence and analysis of H3K4me1 in the midbrain of the Iso-treated group. Data were analyzed by the unpaired t-test and presented as mean ± SEM. **P* < 0.05, ***P* < 0.01, ****P* < 0.001, ns: no significance.

**Supplementary tables**

**Table S1. Demographic and clinical characteristics of PD patients and HCs**

| **Characteristics** |  | **PD (n=70)** | **HC(n=62)** | **p-value** |
| --- | --- | --- | --- | --- |
| Age [mean±SD (range)] |  | 66.87±8.98 | 64.32±12.21 | <0.01 |
| Sex (M/F) |  | 42/28 | 35/27 | <0.05 |
| Duration of illness (years) |  | 6.19±3.98 | NA | NA |
| MMSE [mean±SD (years)] |  | 27.19±5.67 | NA | NA |
| UPDRS [mean±SD (years)] |  | 39.44±15.84 | NA | NA |
| H&Y stage (number) | I Male | 3 |  |  |
|  | Female | 2 |  |  |
|  | Ⅱ Male | 38 |  |  |
|  | Female | 24 |  |  |
|  | Ⅲ Male | 1 |  |  |
|  | Female | 2 |  |  |
| Drug administration | L-DOPA | 52 | NA | NA |

Data are mean ± SD for age, sex, duration of illness, MMSE, and UPDRS. P-value for comparisons of age using independent t-tests. Abbreviations: PD, Parkinson’s disease; HCs, Healthy controls; MMSE, Mini-Mental State Examination; UPDRS, Unified Parkinson's Disease Rating Scale; H&Y stage, Hoehn &Yahr stage; NA, not applicable.

**Table S2. List of primers used for RT-qPCR.**

| Primer name | Sequence |
| --- | --- |
| *Mtr* | Forward: GCTCTGTGAAGACCTCATCTGG |
|  | Reverse: GAGCCATTCCTCCACTCATCTG |
| *Mtrr* | Forward: CGTGAGGTAGAAGAACTGCTCC |
|  | Reverse: CAGGTGAGGATGAACTGGAGAG |
| *Mthfr* | Forward: TACCTCTCTGGAGAGCCGAATC |
|  | Reverse: GGCTGAGAGTTGATGGTGAGGA |
| *Mthfd2* | Forward: GCGAATGTGCTTGGACCAGTAC |
|  | Reverse: TTTGACCTGCCAGCCACTACCA |
| *Mthfd1* | Forward: AGGAGGTGGATATGCTCAGGTC  Reverse: GCCAGCAAGTTATTAGCGGCAG |
| *Shmt1* | Forward: CTGGAGATGCTGTGTCAGAAGC  Reverse: TGAGGCTCTACCAGGGCAGTAT |
| *Shhmt2* | Forward: TGGAACTCGTCTCCATCACAGC  Reverse: GACTCTACGGAAGTCGTCCTCA |
| *Dhfr* | Forward: GCCACCTCAGACTCTTTGTGAC  Reverse: GACTCTACGGAAGTCGTCCTCA |
| *Tyms* | Forward: GCATGGAGAACTCCAGTACCTG  Reverse: CACTCGTTTGGTTGTGAGCAGAG |
| *Mat1a* | Forward: CCTTCTCTGGAAAGGACTACACC  Reverse: GACAGAGGTTCTGCCACACCAA |
| *Bhmt* | Forward: GAGTTCCTCAGAGCTGGATCGA  Reverse: TCATCAGCCACTTGCCGTGCAA |
| *Bhmt2* | Forward: TGCAGGCTCACCTTATGGTCCA  Reverse: ATCTGGTGGCAACTCTTGGCTC |
| *Gnmt* | Forward: TGGTGATCGACCACCGCAACTA  Reverse: GTCGTAATGTCCTTGGTCAGGTC |
| *Chdh* | Forward: GCGTTGGCAATGCAGATGACCT  Reverse: ACGCTTGCTGAACGTAGACCTC |
| *Ahcy* | Forward: CAGGCTATGGTGATGTGGGCAA  Reverse: CCTCCTTACAGGCTTCGTCCAT |
| *Slc1a1* | Forward: CTTCCTACGGAATCACTGGCT  Reverse: CGATCAGCGGCAAAATGACC |
| *Slc1a2* | Forward: ACAATATGCCCAAGCAGGTAGA  Reverse: CTTTGGCTCATCGGAGCTGA |
| *Slc1a3* | Forward: CTTCCTACGGAATCACTGGCT  Reverse: CGATCAGCGGCAAAATGACC |
| *Slc1a4* | Forward: GGCATCGCTGTTGCTTACTTC  Reverse: CGAGGAAAGAGTCCACTGTCT |
| *Slc1a5* | Forward: CATCAACGACTCTGTTGTAGACC  Reverse: CGCTGGATACAGGATTGCGG |
| *Slc1a6* | Forward: AGCAGCCACGGCAATAGTC |
|  | Reverse: ATGCCAAGCTGACACCAATGA |
| *Glul* | Forward: TGAACAAAGGCATCAAGCAAATG  Reverse: CAGTCCAGGGTACGGGTCTT |
| *Gapdh* | Forward: CCTGGAGAAACCTGCCAAGTA |
|  | Reverse: TCATACCAGGAAATGAGCTTGAC |
| *Pemt-NC* | Forward: UUCUCCGAACGUGUCACGUTT |
|  | Reverse: ACGUGACACGUUCGGAGAATT |
| *Pemt*-siRNA-1 | Forward: CAACCCACUCUUCUGGAAUTT |
|  | Reverse: AUUCCAGAAGAGUGGGUUGTT |
| *Pemt*-siRNA-2 | Forward: CCUGCUAUUCCCUGGGCAUTT |
|  | Reverse: AUGCCCAGGGAAUAGCAGGTT |
| *Pemt*-siRNA-3 | Forward: GGACCUUUCUAGGUGACUATT |
| *Pemt*-siRNA-4 | Reverse: UAGUCACCUAGAAAGGUCCTT  Forward: CGUGGUUGCUCUCCUAUAUTT  Reverse: AUAUAGGAGAGCAACCACGTT |

**Table S3. List of reagents used for Western blot, immunofluorescent, immunohistochemistry, and CUT & Tag.**

| **Target** | **Format** | **Source** | **#Catalog** |
| --- | --- | --- | --- |
| SHMT1 | Rabbit IgG | Novus Biologicals | NBP3-36705 |
| SHMT1 | Rabbit IgG | Biorbyt | orb186122 |
| SHMT1 | Rabbit IgG | ABclonal | A12489 |
| SHMT2 | Rabbit IgG | ABclonal | A1215 |
| PEMT | Rabbit IgG | Biorbyt | orb1880990 |
| GFAP | Mouse IgG | Millipore | MAB360 |
| TH | Mouse IgG | Millipore | MAB318 |
| H3K4me1 | Rabbit IgG | CST | #5326 |
| H3K4me1 | Rabbit IgG | Active Motif | 39299 |
| H3K4me2 | Rabbit IgG | CST | #9725 |
| H3K4me3 | Rabbit IgG | CST | #9751 |
| H3K9me1 | Rabbit IgG | Abcam | ab176880 |
| H3K9me2 | Rabbit IgG | Abcam | ab176882 |
| H3K9me2 | Rabbit IgG | Active Motif | 39375 |
| H3K9me3 | Rabbit IgG | Abcam | ab176916 |
| H3K27me1 | Rabbit IgG | Bioworld Technology | BS7235 |
| H3K27me2 | Rabbit IgG | CST | # 9728 |
| H3K27me3 | Rabbit IgG | CST | # 12158S |
| H3K36me1 | Rabbit IgG | CST | #14111 |
| H3K36me2 | Rabbit IgG | CST | #2901 |
| H3K36me3 | Rabbit IgG | CST | #4909 |
| Histone-H3 | Mouse IgG | Proteintech | 17168-1-AP |
| β-actin | Mouse IgG | Proteintech | 66009-1-Ig |
| GAPDH | Mouse IgG | Proteintech | 60004-1-Ig |
| EAAT2 | Rabbit IgG | Abcam | ab205247 |
| EAAT2 | Mouse IgG | Proteintech | 67083-1-Ig |
| GS | Rabbit IgG | Aladdin | Ab105689 |
| GS | Rabbit IgG | Proteintech | 11037-2-AP |
| Donkey anti-mouse | Alexa Fluor 488 | Invitrogen | A-21202 |
| Donkey anti-rabbit | Alexa Fluor 555 | Invitrogen | A-31572 |
| Donkey anti-rabbit | Alexa Fluor 647 | Invitrogen | A-31573 |
| Goat anti-mouse | Alexa Fluor 555 | Invitrogen | A32727 |
| Goat anti-rabbit | Alexa Fluor 488 | Invitrogen | A-11008 |
| Goat anti-mouse | PolyHRP | HISTOV | PHGM30 |
| Goat anti-rabbit | PolyHRP | HISTOV | PHGR30 |
| Donkey Serum |  | Sigma Aldrich | D9663 |
| Goat Serum |  | Sigma Aldrich | G9023 |
| DAPI |  | Sigma Aldrich | D9542 |
| Nissl Stain Kit (Cresyl Violet Method) |  | Solarbio | G1430 |
| Glutamic Acid (Glu) Content Assay Kit |  | Solarbio | BC1585 |
| Glutamine (Gln) Content Assay Kit |  | Solarbio | BC5305 |
| Mouse Phosphatidylethanolamine N-methyltransferase (PEMT) ELISA Kit |  | Jonlnbio | JL51290 |
| S-Adenosylmethionine ELISA Kit |  | Abcam | ab287822 |
| S-Adenosylmethionine ELISA Kit |  | Biorbyt | orb781945 |
| Phosphatidylcholine (PC) Colorimetric Assay Kit |  | Elabscience | E-BC-K796-M |
