# Supplementary figures and images for: Alternative Pathway for Methyl Supply through the Coupling of SHMT1 and PEMT to Maintain Astrocytic Homeostasis in Parkinson's Disease
Source: Adv Sci (Weinh). 2025 Nov 20;13(7):e16794. doi: 10.1002/advs.202516794 (PMC12866788; doi:10.1002/advs.202516794)

Figure S12A

Representative images

N=1

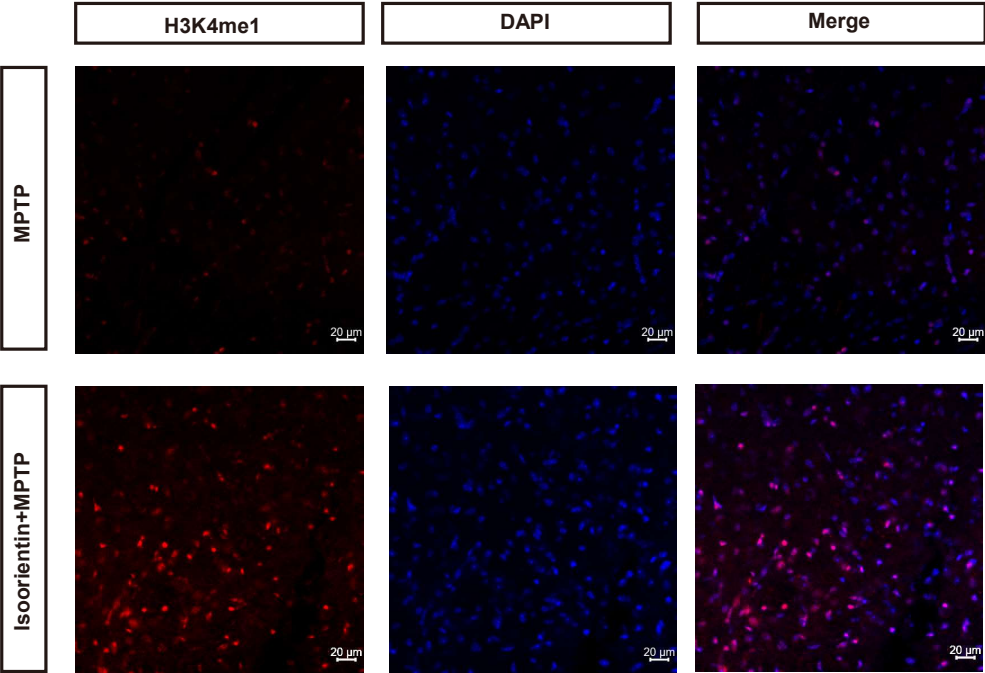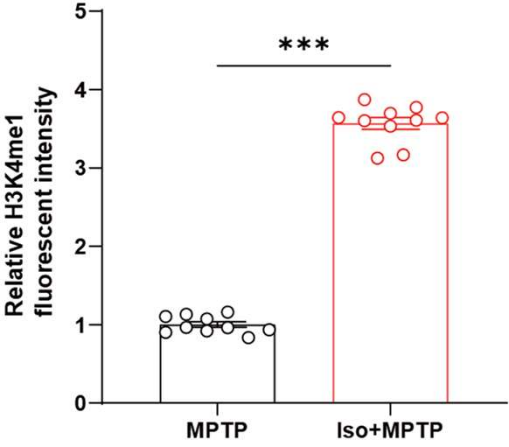

N=2

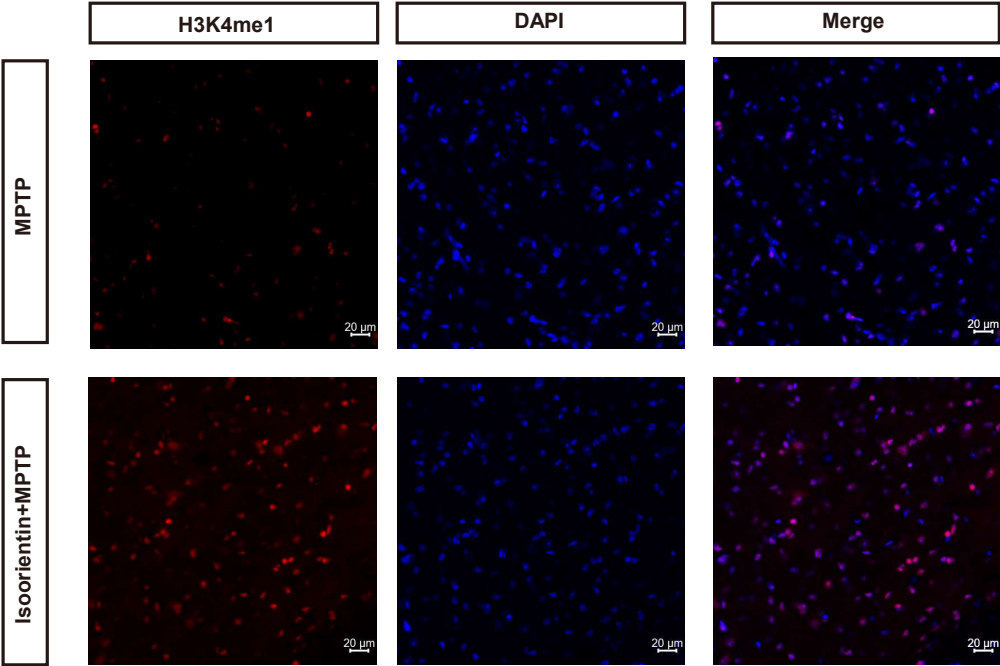

N=3

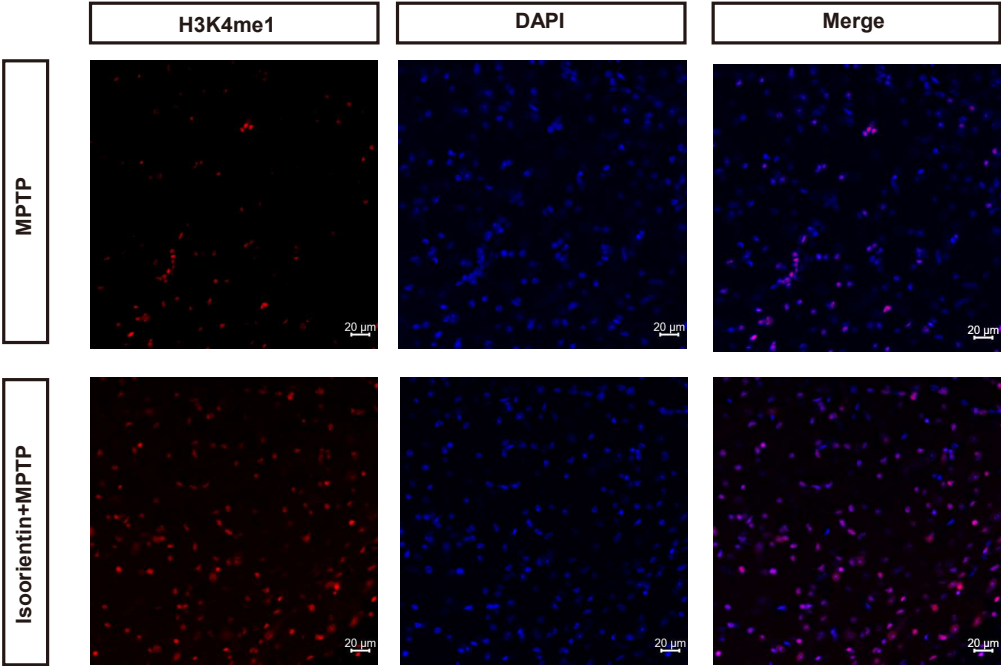

N=4

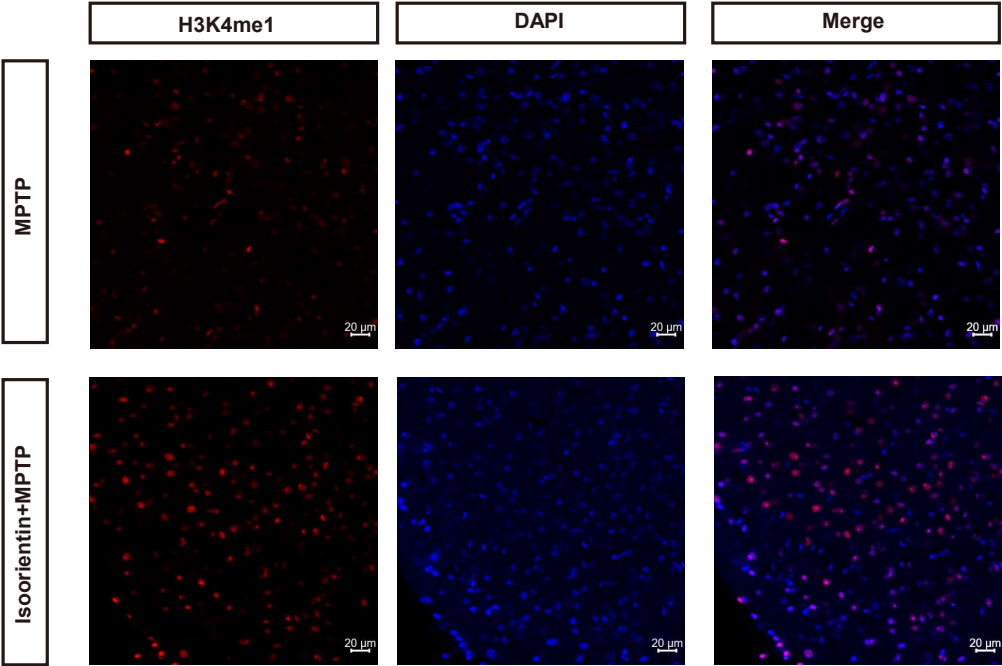

N=5

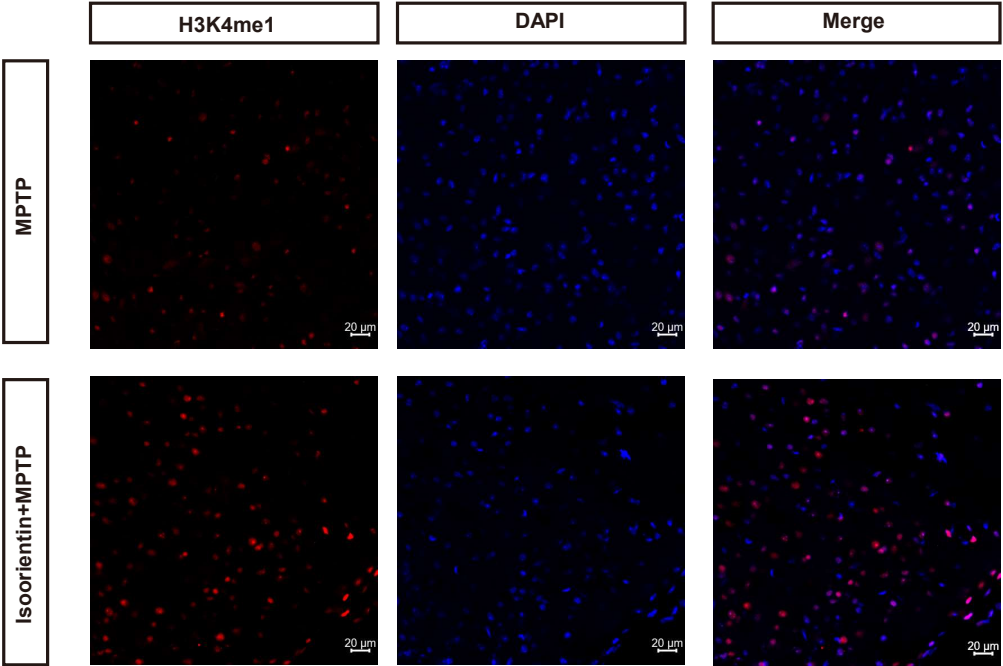

N=6

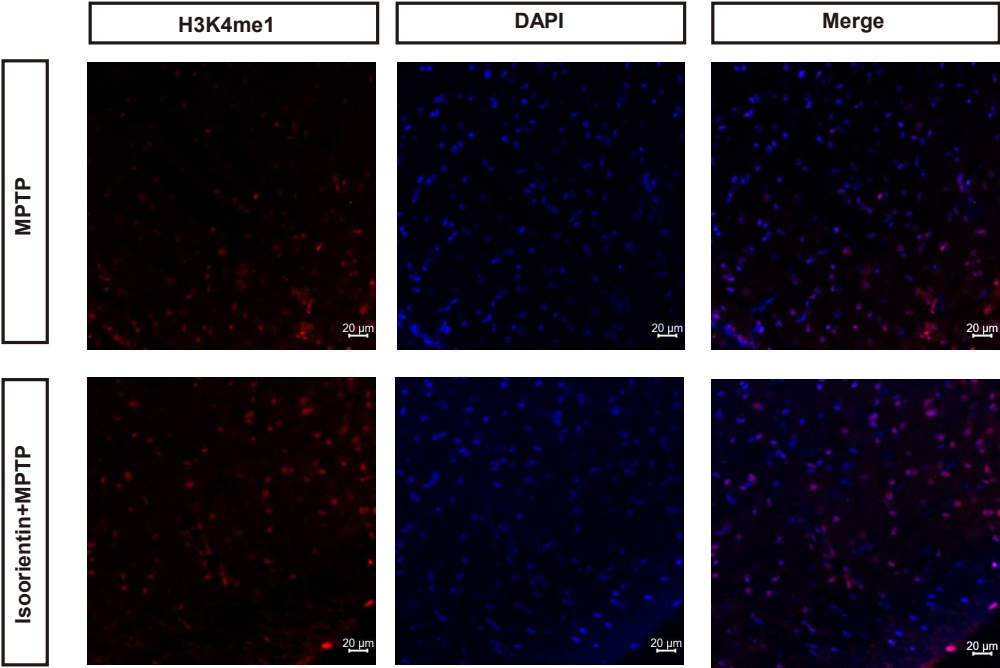

N=7

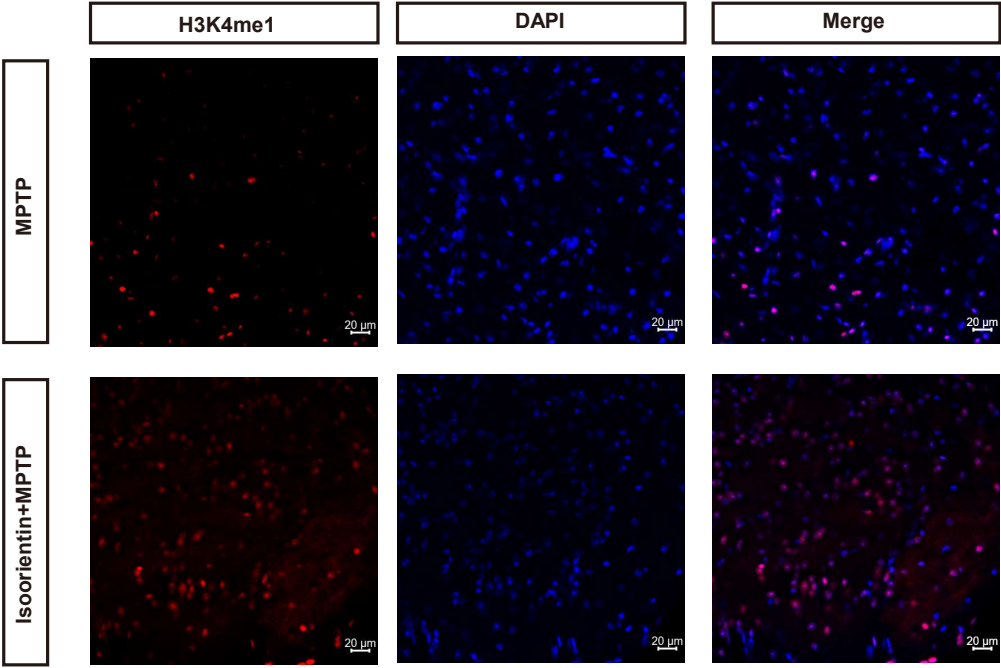

N=8

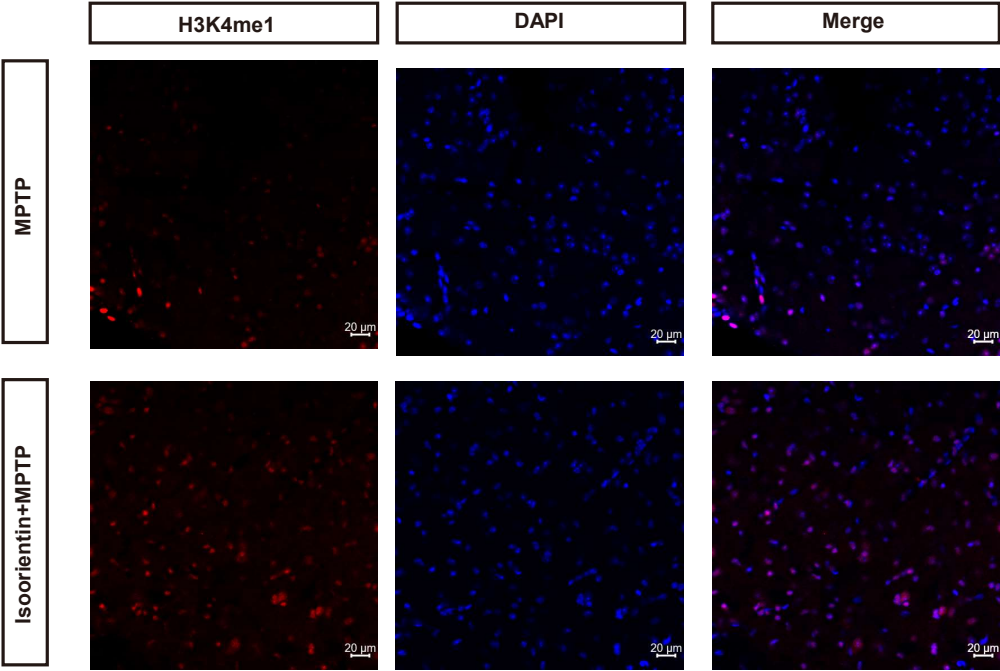

N=9

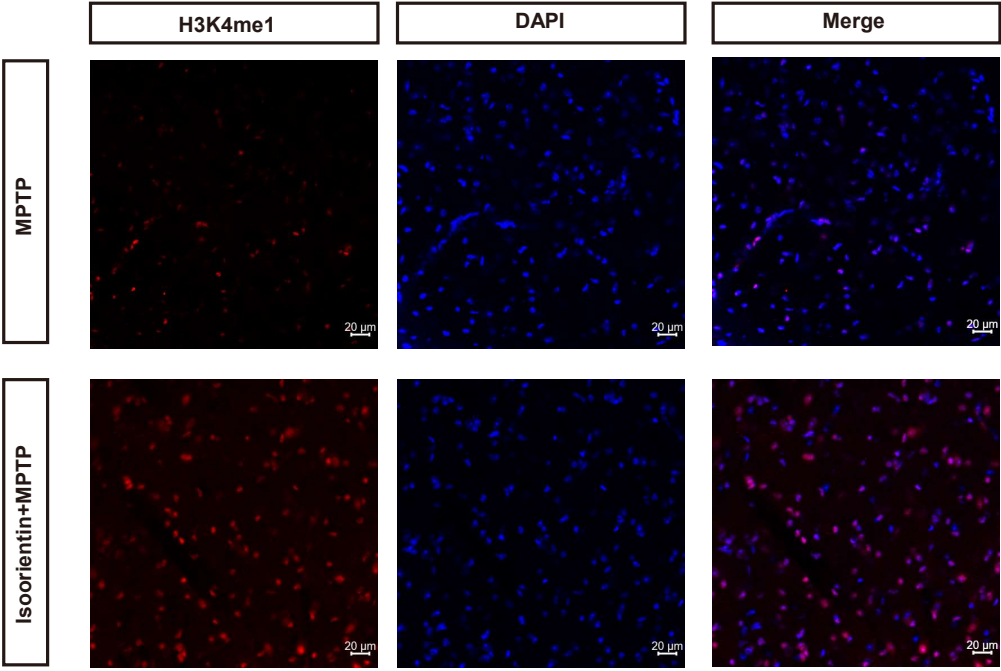

N=10

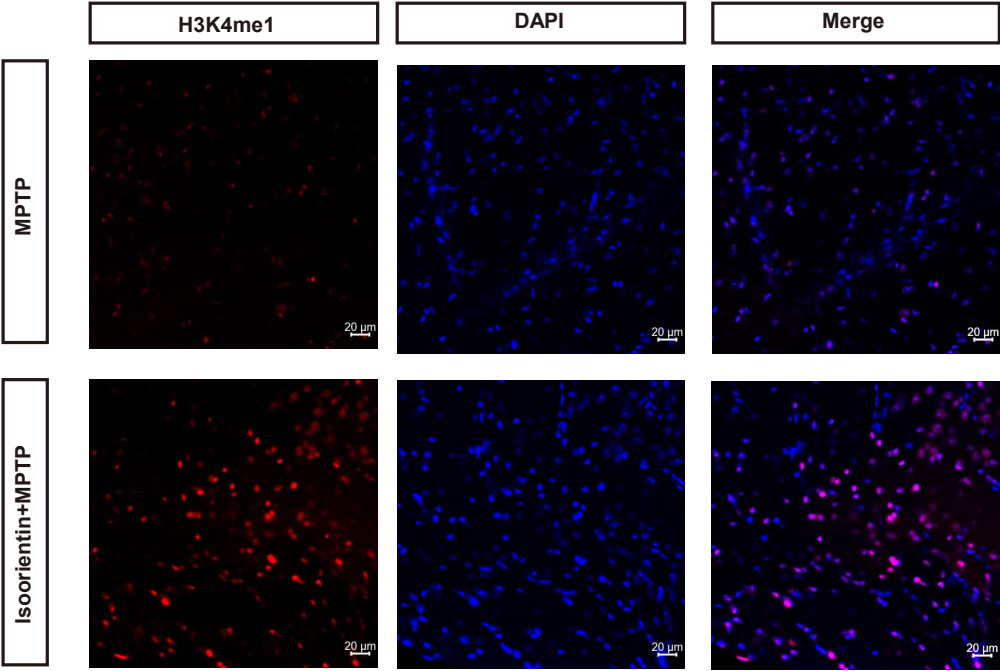

Supplement: Supplementary file 2 — Supporting Information [file ADVS-13-e16794-s001.zip › IF data Figure S12.pdf]
